# The Role of Epigenetics in Placental Development and the Etiology of Preeclampsia

**DOI:** 10.3390/ijms20112837

**Published:** 2019-06-11

**Authors:** Clara Apicella, Camino S. M. Ruano, Céline Méhats, Francisco Miralles, Daniel Vaiman

**Affiliations:** Institut Cochin, U1016 INSERM, UMR8104 CNRS, Université Paris Descartes, 24 rue du faubourg St Jacques, 75014 Paris, France; clara.apicella@inserm.fr (C.A.); camino.ruano@inserm.fr (C.S.M.R.); celine.mehats@inserm.fr (C.M.); francisco.miralles@inserm.fr (F.M.)

**Keywords:** preeclampsia, epigenetics, DNA methylation, non coding RNAs, miRNAs, histone post translational modifications, HOX genes, H19, miR-210

## Abstract

In this review, we comprehensively present the function of epigenetic regulations in normal placental development as well as in a prominent disease of placental origin, preeclampsia (PE). We describe current progress concerning the impact of DNA methylation, non-coding RNA (with a special emphasis on long non-coding RNA (lncRNA) and microRNA (miRNA)) and more marginally histone post-translational modifications, in the processes leading to normal and abnormal placental function. We also explore the potential use of epigenetic marks circulating in the maternal blood flow as putative biomarkers able to prognosticate the onset of PE, as well as classifying it according to its severity. The correlation between epigenetic marks and impacts on gene expression is systematically evaluated for the different epigenetic marks analyzed.

## 1. Introduction

PE affects ~2–5% of the pregnancies. This disease, characterized in the classical definition by hypertension and proteinuria, surging from the mid-gestation at the earliest, is often seen as a two-stage disease, where a placental dysfunction occurs, first without observable symptoms and is followed later by a symptomatic phase from the 20th week of gestation at the earliest. The placenta is central to the disease development [1]. During pregnancy, the cytotrophoblasts (CTs) invade and remodel the structure of the spiral arteries of the myometrium [2]. These changes cause a significant increase in blood flow to the placenta. In a classical vision of the disease etiology, it is said that deep invasion is deficient in preeclampsia [3]. It is generally acknowledged that in preeclamptic pregnancies, placentation is disrupted because the CTs fail to properly invade the myometrium and transform the spiral arteries [4]. This decreases the blood flow and alters the oxygenation of the placenta (causing hypoxia and hyperoxia events), triggering oxidative stress, necrosis and inflammation [5]. In a very stimulating paper, B. Huppertz challenges this classical understanding of PE etiology, by dissociating the defect of deep trophoblast invasion from preeclampsia but rather associating this defect with the Fetal Growth Restriction (FGR) phenotype [6]. In this vision, preeclampsia would rather be caused by a combination of *villous* trophoblast defects (which are not involved in invasion, contrary to *extravillous* trophoblast) and maternal susceptibility. He based his reasoning on the fact that invasion defects are actually not histologically visible in many cases of preeclampsia. This may be connected to mouse models of preeclampsia where no obvious fetal growth restriction occurs, consistently with the fact that invasion is not important in rodent [7]. More accepted than this vision, the same paper strengthens the idea that hyperoxia rather than hypoxia is a major actor of the disease [6,8].

The preeclamptic placenta releases vasoactive molecules, pro-inflammatory cytokines, microparticles and syncytial fragments into the maternal circulation which ultimately cause a systemic endothelial dysfunction [9]. Epigenetics plays an important role in the regulation of the development and physiology of the placenta [10]. Besides, substantial epigenetic alterations, in the preeclamptic placenta and other affected tissues have been described and are likely playing a substantial role in the evolution of the disease [11,12,13,14].

## 2. Epigenetics and Normal Placental Development

### 2.1. Description of the Placenta and Placental Cells

The placenta is a temporary organ connecting the developing fetus to the uterine wall through the umbilical cord, to allow for nutrient absorption, thermal regulation, waste disposal and gas exchange via the mother’s blood supply. In addition, the placenta produces hormones that support pregnancy and it acts as a barrier to fight against internal infection [15].

**Figure 1:**
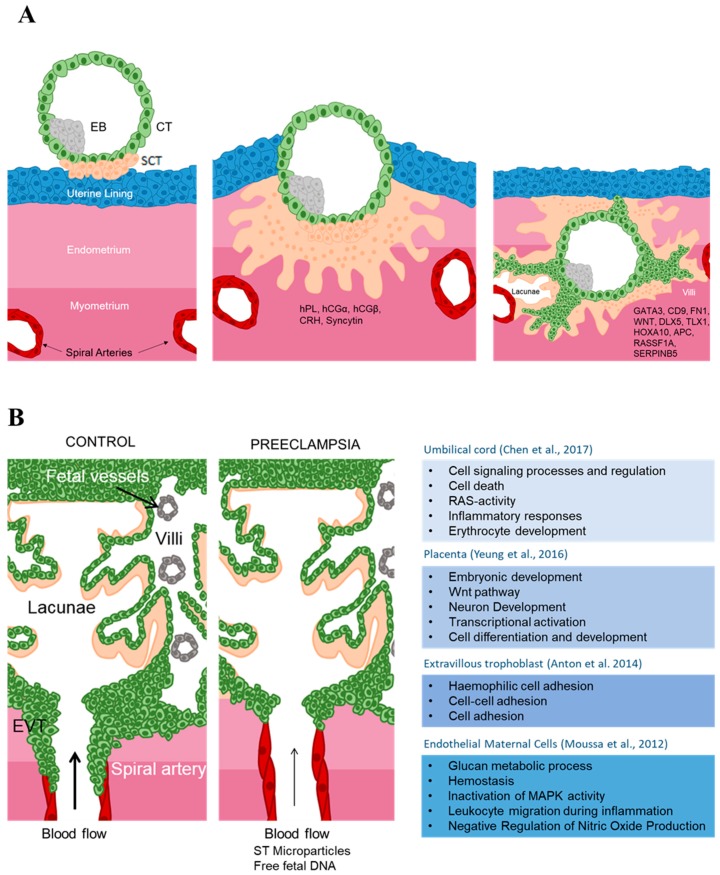
(**A**) Blastocyst implantation and Placenta Development: After recognizing the uterine lining, the blastocyst is formed by the embryoblast (EB) and the cytotrophoblast (CT). The cytotrophoblast starts to differentiate into Synctiotrophoblast (SCT). SCT invades the endometrium towards the maternal spiral arteries located in the myometrium. deregulation of numerous genes is observed [19]. Lacunae develop in the syncytiotrophoblast, which will eventually constitute the intervillous space. Genes upregulated during villi formation are presented on the right figure [20]. Other cytotrophoblasts will invade the maternal spiral arteries by differentiating into Extravillous trophoblast. (**B**) Gene Ontology of genes differentially methylated in PE compared to control samples: (**Left**) in normal pregnancies, extravillous trophoblast (EVT) invades the maternal spiral arteries allowing for an increased blood stream towards the extravillous space. Nutrients cross the placenta, are directed towards the embryonic vessels and collected in the umbilical cord. In PE, decreased invasion of the EVTs induces poor spiral artery remodeling, leading to poor blood flow towards the placenta. Increased amount of microparticles from the syncytiotrophoblast and increased amount of free fetal DNA is observed in the maternal blood. (**Right**) Gene ontology of differentially methylated genes found in PE samples in different tissues affected during pregnancy: Umbilical cord, placenta, EVT, Endothelial Maternal cells (see text for detail).

The human placenta at term has a discoid shape, an average diameter of 15–20 cm, a thickness of 2.5 cm in the center and a weight of about 500 g. Its surfaces are the chorionic plate on the fetus side and to which the umbilical cord is attached and the basal plate facing the maternal endometrium. Between the endometrium and the basal plate there is a cavity filled with maternal blood, the intervillous space, into which branched chorionic villi project. The chorionic villi are the structural and functional unit of the placenta. Their core is made of fibroblasts, mesenchymal cells, endothelial cells, immune cells such as Hofbauer cells (supposed to be macrophage-like) and fetal-placental vessels. The villi are covered by two layers of trophoblasts. The inner layer is composed of villous cytotrophoblasts (vCTs), which are highly proliferative and can differentiate into either outer layer villous syncytiotrophoblasts (SCT), which are in direct contact with the maternal blood or extravillous trophoblasts (EVTs), as shown in Figure 1.

### 2.2. Human Placental Development

The development of the human placenta has been described in detail elsewhere [16,17,18]. Briefly, the blastocyst implants into the uterine endometrium (decidua) via the trophectoderm cells adjacent to the inner cell mass (ICM). From the trophectoderm, the syncytium (SCT) emerges and spreads. Subsequently, CTs proliferate rapidly to form large finger-like projections (villi) that penetrate the entire depth of the SCT. Ultimately, the villi become filled with mesenchyme originated from the extraembryonic mesoderm. This mesenchyme will form fetal blood vessels which connect to the fetal circulation via the umbilical cord. The intervillous space subsequently becomes filled with maternal blood. The vCTs situated at the tips of the anchoring villi proliferate and stratify, forming highly compact cell columns breached only by channels carrying maternal blood toward and away from the placenta (Figure 1). The trophoblast cells within this structure are referred to as EVTs, according to their external location relative to the chorionic villi. EVTs situated close to the decidua, stop proliferating and develop invasive properties. These invasive EVTs migrate deeply into the decidua, where they transform the uterine vasculature in order to supply the placenta maternal blood, a critical step in establishing uteroplacental circulation. As pregnancy progresses, the number of vCTs decreases and few is observable at term underneath the SCT.

### 2.3. Epigenetics Mechanisms in Placental Development

Epigenetic mechanisms are involved in the regulation of gene expression both during development and in differentiated tissues [21,22]. These mechanisms include DNA methylation, histone modifications and biogenesis and action of noncoding RNAs (ncRNAs). They regulate gene expression by modulating the accessibility to DNA of transcription factors and other regulatory proteins. In addition, ncRNAs also regulate gene expression at a post-transcriptional level. Epigenetic mechanisms are essential for cellular differentiation and therefore development, as summarized in Table 1

#### 2.3.1. DNA Methylation

The best studied epigenetic mechanism in the placenta is DNA methylation, the covalent addition of a methyl group to a cytosine, usually in the context of cytosine-phospho-guanine (CpG) dinucleotides. Several reviews have been dedicated to the role of this mechanism in placental development [10,12,61]. Also, several high-throughput analyses have been performed to analyze the methylation epigenetics of the developing placenta (Table 2, Supplementary Table S1 for the details).

##### Differentiation of Stem Cells

Contrary to mice, a Trophoblast Stem Cell (TSC) population has not yet been clearly identified in humans, thus limiting our capacity to study the role of DNA methylation in the early stages of trophoblast differentiation. A recent study has addressed this question using a side-population trophoblasts, a candidate human TSC [55], isolated from first trimester placenta. The comparison of the methylomes of this side-population trophoblasts and the methylomes of vCTs and EVTs all isolated from the same first trimester placenta, showed that each population had a distinctive methylome [56]. In comparison to mature vCTs, side-population trophoblasts, showed differential methylation of genes and miRNAs involved in cell cycle regulation, differentiation and regulation of pluripotency. In addition, the comparison of the methylomes and transcriptomes of vCTs and EVTs revealed the methylation of genes involved in epithelial-mesenchymal transition (EMT) and metastatic cancer pathways, which could be involved in the acquisition of the invasive capacities of the EVTs. However, this study, as many others, failed to establish a systematic correlation between hypermethylation of the genes and downregulated expression. Therefore, the authors conclude that although CpG methylation is involved in the trophoblasts differentiation, it cannot be the only regulatory process.

##### Regulation of Homeotic Genes

Several studies have identified and established the importance of the transcription factors of the homeobox gene family (HOX) in the development of human placenta [82,83,84,85,86]. Most HOX genes have been found stably hypo-methylated throughout gestation, suggesting that DNA methylation is not the primary mechanism involved in regulating HOX genes expression in the placenta. However, these genes show variable methylation patterns across gestation, with a general trend towards an increase in methylation over gestation. Three genes (*TLX1, HOXA10* and *DLX5*) present slightly increased methylation while their mRNA expression decreases throughout pregnancy, supporting a role for DNA methylation in their regulation [46]. Down-regulation of these genes using siRNAs specific for *DLX5*, *HOXA10* and *TLX1* in primary trophoblasts leads to loss of proliferation and to an increase in mRNA expression of differentiation markers, such as *ERVW-1*. This suggests that loss of these proteins is required for proper SCT development [46].

##### Placental Development and Cancer Pathways

The early steps of placentation are reminiscent of the invasive properties of malignant tumors. Studies on DNA methylation in cancer cells and placental cells have highlighted similarities in their epigenomes, particularly, a widespread hypomethylation throughout the genome and focal hypermethylation at CpG islands. Hypomethylation within the placenta is not uniform but occurs in large domains (>100 kb) called partially methylated domains (PMDs) which are regions of reduced DNA methylation that cover approximately 40% of the placental genome [51]. PMDs are unique to a few different tissue types that include the placenta, cultured and cancer cells [50,51,87]. Placental genes within PMDs tend to be tissue-specific and show higher promoter DNA methylation and reduced expression as compared with somatic tissues [51]. A genome-wide comparison of DNA methylation changes in placental tissues during pregnancy and in 13 types of tumor tissues during neoplastic transformation revealed that megabase-scale patterns of hypomethylation distinguish first from third trimester chorionic villi in the placenta [52]. These patterns mirror those that distinguish many tumors from the corresponding normal tissues. The genomic regions affected by this hypomethylation encompass genes involved in pathways related to EMT, immune response and inflammation, all of them associated to cancer phenotypes. Moreover, the authors observed that hypomethylated blocks distinguish vCTs before 8–10 weeks of gestation and after 12–14 weeks of gestation. The analogy between early placentation and malignant tumors at the epigenetic level is further stressed by studies analyzing the methylation status of the promoters of several tumor suppressor genes (RASSF1A, SERPINB5 also known as APC and Maspin, respectively) in the developing placenta and human choriocarcinoma cell lines (JAR and JEG3) [25,49]. These studies show that promoter DNA-methylation regulates the expression of these tumor suppressor genes which in turn affects the migration and invasive capacities of the trophoblastic cells (As summarized in Table 1).

#### 2.3.2. Non-coding RNAs and Epigenetic Regulation of Placenta Development

##### Definition

A non-coding RNA (ncRNA) is defined as an RNA molecule that is not translated into a protein. Classes of non-coding RNAs include transfer RNAs (tRNAs) and ribosomal RNAs (rRNAs), small RNAs such as microRNAs (miRNAs), siRNAs, piRNAs, snoRNAs, snRNAs, exRNAs, scaRNAs and the long ncRNAs [88]. The role of these molecules in placental development, physiology and pathology has been recently reviewed in detail [89]. Here we will discuss solely the role of miRNAs and long ncRNAs in the epigenetic control of placental development.

##### MiRNA and Normal Human Placental Development

The miRNAs are single stranded RNA molecules of 19–24 nucleotides, which act primarily by degrading mRNA transcripts or inhibiting translation of miRNA in to proteins [90]. To date, more than 2000 human miRNAs have been discovered, which appear to regulate 50% of human RNAs [91]. A large number of miRNAs detected in the placenta are expressed from a gene cluster located on chromosome 19 (C19MC) [92,93]. This cluster includes 46 intronic miRNA genes that express 58 miRNA species. These miRNAs are primate-specific, and they are expressed almost exclusively in the placenta (and are thus termed trophomiRs). In the human placenta, the expression of C19MC miRNAs is detected as early as 5 weeks of pregnancy and the expression gradually increases as pregnancy progresses [94]. An imprinted, paternally expressed, CpG-rich domain has a regulatory role in C19MC expression [95]. This DMR, is hypermethylated in cell lines that do not express C19MCs [96]. The C19MC region contains genomic transposable elements called “Alu repeats”, which have been implicated in recombination and gene duplication events. Because of their sequence complementarity it has been proposed that several C19MC miRNAs could be responsible of the targeting and degradation of transcribed Alu elements. Also, the C19MC miRNAs are expressed in embryonic and in stem cells but their expression drops considerably when these cells differentiate, which may indicate a role in the maintenance of an undifferentiated state [97,98,99,100,101]. Several members of the C19MC cluster are expressed at much higher levels in vCT compared with EVTs and overexpression of the C19MC cluster results in reduced migration of the extravillous trophoblast line HTR8/SVneo [45]. The chromosome 14 miRNA cluster (C14MC) is another miRNA cluster that is expressed in the placenta [102]. This cluster includes the miRNAs: miR-127, miR-345, miR-370, miR-431 and miR-665. These miRNAs have been involved in the regulation of the immune suppressive, anti-inflammatory response and also in the regulation of the ischemia/hypoxia response [103]. The expression of the C14MC members generally declines during pregnancy [104].

The miR-675 is expressed from the first exon of the H19 long non-coding RNA. Up-regulation of miR-675, which is controlled by the stress-response RNA-binding protein HuR, restricts murine placental growth. Deficiency of H19, promotes placental growth and miR-675 overexpression decreases cell proliferation, likely through targeting Igf1R [105]. Consistent with these findings, the expression of miR-675 rises toward the end of murine pregnancy, when placental growth decelerates. In addition, miR-675 restricts proliferation in JEG3 cells, likely through binding to the nodal modulator 1 (NOMO1) protein [44].

Several other miRNAs are likely involved in placental development by inhibiting genes associated to regulation of trophoblast fate, invasion and proliferation (Let-7a, miR-377, miR-145, members of the miR-17_92 cluster, members of the miR-106a_363 and miR-106b_25 clusters, miR-155, miR-34, miR-141-3p and miR- 200a-3p) [106,107]. As additional examples of regulation, mir-431inhibits invasion of trophoblast cells by targeting the ZEB1 gene [108], miR-106a~303 inhibits trophoblast differentiation by targeting hCYP19A1 and hGCM1 [43], miR-34 targets SERPINA3, a key gene in a variety of biological processes and highly deregulated in placental diseases [41].

These miRNAs regulate diverse processes such as trophoblast physiology, proliferation and invasion (some mentioned in Table 1 and reviewed in Reference [107]).

##### lncRNA and Normal Human Placental Development

Long non-coding RNAs (lncRNAs) are RNAs greater than 200 nucleotides in length that do not encode a protein product. They are expressed with cellular and temporal specificity and have been involved in many cellular events, including the regulation of gene expression, post-transcriptional modifications and epigenetic modifications, imprinting and X-chromosome inactivation [109]. They act as scaffolds (binding other RNAs or proteins), signals and antisense decoys and engage in transcriptional interference. Usually a single lncRNA has multiple functions. The function of lncRNAs in placental development is poorly understood, mostly inferred from studies on placental pathologies. Nevertheless, lncRNAs have been involved in a number of critical trophoblast functions, from proliferation, invasion and migration, to cell cycle progression [110]. H19 was one of the first lncRNAs to be discovered [111]. H19 is located within a large imprinted domain on chromosome 11, at ~100 kb downstream of IGF2. H19 and IGF2 are reciprocally imprinted that is, for H19 only the maternal allele is expressed, while for IGF2, only the paternal allele is expressed [112]. H19 expression could be regulated by PLAGL1, a zinc finger transcription factor, in the human placenta [113]. Two major functions have been described for H19, specifically as a modulator for binding small RNAs and proteins [114] and as a source of the miRNA mir-675 (see above). H19 has variable levels of biallelic expression in the placenta (reports suggest between 9% and 25% expression occurs from the imprinted allele) until 10 weeks of gestation by which time H19 expression is mostly restricted to the maternal allele [115]. H19 expression is restricted to intermediate and vCT and is not found within SCTs in the human placenta. H19 down-regulation in trophoblast cells leads to inhibition of proliferation and apoptosis [116]. Many other lncRNAs have been involved in placental development, including lincRNA SPRY4-IT1, MIR503HG, LINC00629, MEG3, MALAT1, RPAIN and TUG1 [31,32,33,34,35,36,37]. The study of the expression of these lncRNAs during placental development and the manipulation of their expression in vitro in choriocarcinoma cell made it possible to infer their possible function in the context of placental development (Table 1).

#### 2.3.3. Histone Modifications in the Developing Placenta

Histone modification is the process of modification of histone proteins by enzymes, including post-translational modifications, such as methylation, acetylation, phosphorylation and ubiquitination. Histone modifications participate in gene expression regulation by modulating the degree of chromatin compaction [117].

Our knowledge concerning the role of histones modification in human placentation is scarce and refers mostly to studies in mice. Methylation frequently occurs on histones H3 and H4 on specific lysine (K) and arginine (A) residues. Histone lysine methylation can lead to activation or to inhibition, depending on the position in which it is located. For instance, H3K9, H3K27 and H4K20 are considered as important ‘inactivation’ markers, that is, repressive marks, because of the relationship between these methylations and heterochromatin formation. However, the methylation of H3K4 and H3K36 are considered to be ‘activation’ marks [118,119].

The heterochromatin methylation marker H3K27me3 was found to be highly active in vCT. That was explained by rapid and transient repression of genes at the time of SCT formation. SCTs nuclei were also found enriched for H4K20me3 [30]. However, this report contrasted with another study reporting that the CTs were enriched with H3K4me3 and that the SCTs were transcriptionally activated by the chromatin marker H3K4me2, which co-localized with active RNAP II in the majority of SCT nuclei [29]. In mouse and other mammals, H3 arginine methylation predisposes blastomeres to contribute to the pluripotent cells of the ICM, which appears to require higher global levels of H3 arginine methylation than the TE/trophoblast lineage [120]. Nevertheless, these lower modification levels in the trophoblast lineage are indispensable for normal placental development.

Acetylation, which in most cases occurs in the N-terminal conserved lysine residues, is also an important way to modify the histone proteins, for example, acetylations of lysine residues 9 and 14 of histone H3 and of lysines 5, 8, 12 and 16 of histone H4 by Histone Acetylases (HATs). Acetylation is generally associated with the activation or opening of the chromatin. On the contrary, de-acetylation of the lysine residues by histone deacetylases (HDACs) leads to chromatin condensation and inactivation of gene transcription. Oxygen (O_2_) concentrations strongly influence placental development partially through modifications of the histone methylation codes. Initially, the gestation environment is hypoxic and O_2_ concentration increases during development. Hypoxia-inducible factor-1 (HIF-1), consisting of HIF-1α and ARNT subunits, activates many genes involved in the cellular response to O_2_ deprivation [121]. HIF-1 is also known to recruit and regulate HDACs [122,123]. Moreover, HIF-1 has been found to bind specific sites on the promoter of the H3K9 demethylases thereby inducing their expression. In particular, it induces JMJD1A and JMJD2A that remove dimethyl marks on H3K9me2, JMJD2B [124,125] which removes trimethyl marks (H3K9me3) and more weakly JMJD2C which converts H3K9me3 to me2 [126]. Studies in rodents have shown that HIFs have important roles in the regulation of TSCs differentiation by integrating physiological, transcriptional and epigenetic inputs. Thus, the crosstalk between HIF and the HDACs is required for normal trophoblast differentiation [123,127].

Another example of histone modification during placentation, is the acetylation of histones H2A and H2B by the CREB-binding protein (CBP). CBP acts as an acetyltransferase that decreases the EMT and invasiveness of murine TSCs while maintaining the properties of stem cells [28].

Trophoblastic fusion depends on the regulation of GCMa activity by HATs and HDACs. Human GCMa transcription factor regulates expression of syncytin, which in turn mediates trophoblastic fusion. It has been demonstrated that CBP-mediated GCMa acetylation underlies the activated cAMP/PKA signaling pathway that stimulates trophoblastic fusion [27]. Human pregnancy-specific glycoproteins (PSG) are the major secreted placental proteins expressed by the SCTs and represent early markers of cytotrophoblast differentiation. Pharmacological inhibition of HDACs in JEG-3 cells up-regulated PSG protein and mRNA expression levels. This correlated with an increase in the amount of acetylated histone H3 associated with PSG promoter [26]. Combined acetylation at H3K9 and H3K4 methylation also activates Maspin, a tumor suppressor gene which is negatively correlated with human trophoblasts motility and invasion [24,25]. The invasive capacity exhibited by EVTs is attributed in part to the extracellular matrix degradation mediated by matrix metalloproteinases (MMPs) such as MMP-2 and MMP-9. Differential expression of these MMPs and their tissue inhibitors (TIMPs) has been associated to histone H3K9/27me3 [23].

#### 2.3.4. Imprinting and Placental Development

##### Placentation and the Materno-Fetal Conflict

Pregnancy in Eutherian mammals is an immunological challenge as reviewed recently [128]. To note, an ancestral inflammatory response in pregnancy and parturition also exist in marsupials (metatherians), as recently observed [129,130]. Other mechanisms are equally conserved in the formation of the placenta, in particular the fusion mechanisms of cytotrophoblasts into syncytiotrophoblasts that are mediated by retroviruses, in eutherians as well as in metatherians [131].

Once the placenta is formed, it will allow nutrients to transit from the mother circulation to the fetal circulation. In the context of the maternal-fetal conflict hypothesis, tightly regulating the placentation process and limiting placental growth is crucial for the mother survival. The genes controlling this regulation are expected to be found different between viviparous and non-viviparous species. For this, mammals appear as an excellent model as a group of ~4500 species divided into egg-laying animals (prototherians, Platypus and Echidnaes, 5 species), animals with a short-lived placenta (metatherians, Marsupials ~250 species) and viviparous species with a long-lived placenta (eutherians, i.e., all the other mammals, where gestation length can be up to 22 months in the African elephant). One major difference found between the genome of placental species and non-placental species of mammals is the presence of imprinted genes only in the first group.

##### Definition of Imprinted Genes and Links with Viviparity

Imprinted genes are genes that are expressed from either the maternal or the paternal allele, mainly through differentially methylation mechanisms. Their existence leads to dramatic phenotypic differences in animal hybrids according to the sense of the cross. For instance, interbreeding of lions and tigers results in two morphologically different animals, if the male is the lion or the male is the tiger, leading to a liger or a tigon, respectively [132]. While the tigon has a size like that of its parents, the liger is the largest existing felid (up to >400 kg) and several hypotheses have been raised to explain this fact, mostly connected to the existence of imprinted genes. Experimentally, in the 80s, Solter and Surani carried out nuclear transfer experiments that demonstrated in mice the necessity of a paternal and maternal genome to foster healthy development [133]. Androgenetic embryos lead to the production of a hypertrophic placenta while gynogenetic embryos had a very small placenta and a stunted embryo. Similarly, in humans, development from two paternal genomes leads to hydatiform moles, where the placenta is composed of grapelike vesicles, whereas parthenogenic development leads to the apparition of teratomas [134].

As far as we know today, imprinting is closely associated to viviparity. The sequencing of the platypus genome in 2008 [135] revealed syntenic regions that are relatively well conserved with the eutherian and marsupials, albeit no evidence of imprinted gene can be found in Monotremes. This may be since acquisition of imprinting in a species seems to be associated to the progressive acquisition of CpG islands (besides other mechanisms, such as chromosome translocations or retrotransposons insertions), that appear absent from the platypus genome [136,137]. In marsupials (metatherians), where the placenta is short-lived, the number of imprinted genes is more limited than in eutherian mammals. Two imprinted regions are well conserved between metatherians and eutherians such as the PEG10 and the H19-IGF2 regions [135]. Similarly, an exhaustive analysis of the transcriptome of chicken failed to identify imprinted genes, while allele specific expression does exist [138,139]. The evidence collected therefore strongly links these genes with the placenta presence. Besides, imprinted genes may have a strictly paternal or strictly maternal expression. Series of invalidation experiments in mice indicated that paternal genes tend to increase placental growth while maternal genes tend to limit this growth [140].

##### Example of the H19-IGF2 Cluster; Cross Species Conservation of Imprinted Genes

A well-known example of this is the H19-IGF2 cluster localized distally at 11p15.5 in humans and 7qF5 in mice. In both species, the structure of the locus is conserved (about 100 kilobases separating the two genes, with differentially methylated regions inside IGF2 and nearby H19). An IMC (Imprinting Control Region), located 3 kb from the starting point of H19 has also been identified, with seven binding sites for the ZNF transcription factor CTCF. H19 is expressed exclusively form the maternal allele, while IGF2 is expressed from the paternal allele. In mice, a placental specific promoter of Igf2 was discovered. The selective invalidation of this promoter [141] leads to a strong decrease of placental development and placental growth. By contrast, the invalidation of H19, leads to placental and fetal overgrowth [142]. Amongst other imprinted genes that affect placental and fetal growth besides H19 and IGF2 are paternally expressed genes, generally identified in mice (Peg1, Peg3, Rasgrf1, Dlk1) and maternally expressed genes (Igf2r, Gnas, Cdkn1c, Grb10).

Interestingly, in mice, the decoy receptor of Igf2, Igf2r is imprinted and with a maternal profile of expression. In humans, surprisingly, the imprinting status of IGF2R seem to be erratic, polymorphically imprinted according to the human individual analyzed. This was first published in 1993 [143] that showed that 2 out of 14 fetuses had an exclusive expression from the maternal allele. Recently it was shown that IGF2R is duly imprinted in macaques [144], showing that even in primates, the imprinting status can vary between relatively close species. Overall, it appears that many placental imprinted mouse genes are biallelic in their expression in humans [145]. Reciprocally, in a study aiming at identifying novel imprinted genes in the human placentas, we compared variants of the placental DNA versus those of cDNAs from the same placentas using SNP microarrays [146,147]. In addition to four known imprinted genes (IPW, GRB10, INPP5F and ZNF597), we could identify 8 novel imprinted genes in the human placentas (ZFAT, ZFAT-AS, GLIS3, NTM, MAGI2, ZC3H12C, LIN28b and DSCAM). Using a mouse cross allowing the following of the allelic origin, we found an astonishing variegation of the imprinting status: only Magi2 was imprinted in the mouse species.

Imprinted genes may have a general impact on the global methylation status of the placenta. For instance, recently a polymorphism located at the IGF2/H19 locus was shown associated to placental DNA methylation and birth weight in association with Assisted Reproductive Technologies usage [148].

Imprinted genes deregulation in the placenta is linked to placental diseases, as reviewed in References [149,150]. In a recent study, Christians and coworkers, analyzed a list of 120 imprinted genes in relation with global expression of 117 placental samples, including PE and Intra Uterine Growth Restriction (IUGR) cases [151]. The authors identified a significant correlation between birth weight and the expression level of imprinted genes but without significant differences between paternally versus maternally expressed genes. Imprinted genes were also more heavily deregulated in preeclampsia than other genes and in this case paternally expressed genes were down-regulated, while maternally expressed genes were up-regulated. The trend was similar for IUGR. Interestingly, the two human-specific microRNA clusters (C19MC and C14MC), both appear to be imprinted (paternally and maternally expressed) for C19MC and C14MC, respectively, clusters that have been duly studied by the team of Yoel Sadovsky [45,89,152]. Recently, we identified duplication in the 19q13.42 imprinted region encompassing the C19MC cluster [153], from a male 26 weeks fetus with severe IUGR, suggesting that a double dose of the miRNA could contribute to the disease. This suggests links between miRNA regulation, imprinting status and the putative consequences for fetal health and growth.

## 3. Epigenetic Alterations in Preeclampsia

### 3.1. DNA Methylation Alterations in Preeclampsia

Anomalies of DNA methylation in preeclampsia have been analyzed from different cellular sources. Besides the analysis of placental cells, investigators have analyzed circulating maternal blood cells or cell-free DNA, as well as maternal endothelial cells (much less accessible, though) and cord-blood white blood cells (of fetal origin). A list of genes of which methylation was found altered is presented as Table 3.

**Figure 2:**
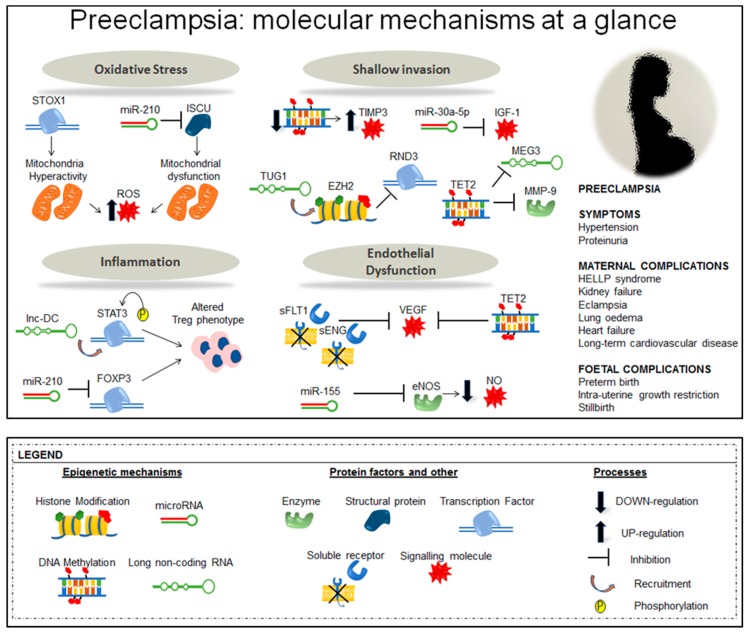
Overview of the molecular mechanisms at play in preeclampsia. Annotations: eNOS = Endothelial Nitric Oxide Synthase; EZH2 = Enhancer of Zeste Homolog 2; FOXP3 = Forkhead box P3; IGF-1 = Insuline-like Growth Factor 1; ISCU = Iron-sulfur cluster; Lnc-DC = Long non-coding RNA DC; miR-30a-5p = microRNA 30a-5p; miR-155 = micro-RNA 155; miR-210 = microRNA 210; MMP-9 = *Matrix Metalloproteinase-9;* NO = Nitric Oxide; RND3 = Rho Family GTPase 3; ROS = Reactive Oxygen Species; sENG = Soluble endoglin; sFLT1 = Soluble fms-like tyrosine kinase *receptor*-1; STAT3 = Signal transducer and activator of transcription 3; STOX1 = Storkhead Box 1; TET2 = Tet methylcytosine dioxygenase 2; TIMP3 = TIMP Metallopeptidase Inhibitor 3; TUG1 = long non-coding RNA taurine-upregulated gene 1; VEGF = Vascular Endothelial Growth Factor.

A summary of epigenetic mechanisms at work in PE is shown in Figure 2.

#### 3.1.1. Methylation Alterations in the Preeclamptic Placenta

##### Common Alterations of Gene Expression in PE are Associated to Methylation Alterations

Numerous studies revealed altered expression of various genes in the pathological placentas (as synthesized previously [154]). These alterations of gene expression are partly explained by the existence of epigenetic deregulations. In PE, numerous methylation deregulations have been found in the pathological compared to control placentas, some studies (but not all) taking into account the gestational age, a recurrent issue when normal and pathological placentas are compared, for which there is often a more than 6 weeks difference [155,156,157,158,159,160]. The different techniques used to analyze methylation globally are presented in a previous review [161]. These epigenetic changes probably originate from the abnormal placental environment in PE (or IUGR), characterized by alternations of low oxygen tension and hyperoxia. As mentioned above, hypoxia *per se* induces the expression of the Hypoxia-Inducible factor (HIF1α), which binds to Hypoxia Responsive Element activating the transcription of various genes related with angiogenesis and metastasis-associated genes [162]. Overall, abnormal oxygen signaling in the placental context leads to increased concentrations of Oxygen Reactive Species (ROS) [163]. Oxidative stress may drive an accelerated ageing of trophoblast cells, which could be key to understand the origin of placental disorders. Indeed, several studies emphasized alterations of telomere length (a mark of ageing) in preeclamptic pregnancies, with a drastic augmentation of short telomeres in PE, especially in Early Onset PE (EOPE) [164,165,166]. This senescence may be induced by alterations of the management of oxidative stress [167,168,169]. The accelerated transformation of vCTs into SCTs will lead to a decrease life expectancy of the placenta and an alteration of its capacity to bring the gestation harmoniously to its normal term.

It is well known that persisting environmental variations induce changes in the epigenetic marks, including DNA methylation. These marks can either be mere biomarkers or participate actively in regulating genes to overcome the changing environmental conditions (although gene expression changes are often disconnected from methylation alterations).

Overall, several of the genome-wide studies showed that the methylation profiles differ between early and late onset of preeclampsia (EOPE and LOPE), suggesting a different etiology between these two types of PE [170,171,172,173]. EOPE shows more pronounced genome-wide hypermethylation changes than LOPE, probably since it is caused by earlier alterations allowing the epigenetic reprogramming to install earlier, in reason of the earlier cellular stress [171,174].

Using the Illumina Methylation 450 BeadChip Array, Yeung and coworkers, identified 303 differentially methylated regions in PE, 214 hyper and 89 hypomethylated, after adjusting for gestational age. The genes located nearby or encompassing hypermethylated regions were enriched in gene-ontology (GO) terms such as “ATP transport”, in KEGG pathways, such as “steroid hormone biosynthesis”, “cellular senescence” and Reactome pathways, such as “Vpr-mediated induction of apoptosis by mitochondrial outer membrane (SLC25A6 and SLC25A4)”. The annotation of clusters also revealed an alteration of clusters of homeobox genes, (especially HOXD genes), Wnt2 cell signaling; fertilization and implantation genes; reactive oxygen species signaling (NOX5) and cell adhesion (ALCAM) genes [158]. Amongst the most recent studies, Leavey and coworkers used a novel approach based upon bioinformatics to sort 48 human PE samples through their transcriptome profile before subjecting them to methylation analysis, using the Illumina Human methylation450K array. This made it possible to divide the preeclamptic cases into two groups associated to abnormal methylation marks nearby ‘immunological’ genes or more ‘canonical’ EOPE cluster, with for instance abnormally methylated CpG in FLNB, COL17A1, INHBA, SH3PXD2A, as well as in the gene body of FLT1 [160].

In 2015, the study of Zhu and coworkers [175] was the first to analyze simultaneously methylation and hydroxymethylation in the PE placentas. Hydroxymethylation results from the hydroxylation of methyl-Cytosine is a first step towards the active demethylation of DNA through the action of Ten+Eleven Translocation enzyme (TET) proteins, and could play an important role in gene expression regulation [176]. The authors showed that the methylation level is higher in gene promoters and gene bodies in PE versus control placenta. Surprisingly most of the clustering of the genes that were altered, either by methylation or by hydroxymethylation were associated with nervous system development, neurotransmitters, neurogenesis, which are presumably not relevant in a non-neural tissue as the placenta. Nevertheless, positive regulation of vasoconstriction was also enriched as a GO term, as well as regulation of nitrogen compounds, two pathways that have a clear biological sense in terms of placental diseases pathophysiology (association with vascularization and with the modulation of oxidative/nitrosative stresses).

**Figure 3:**
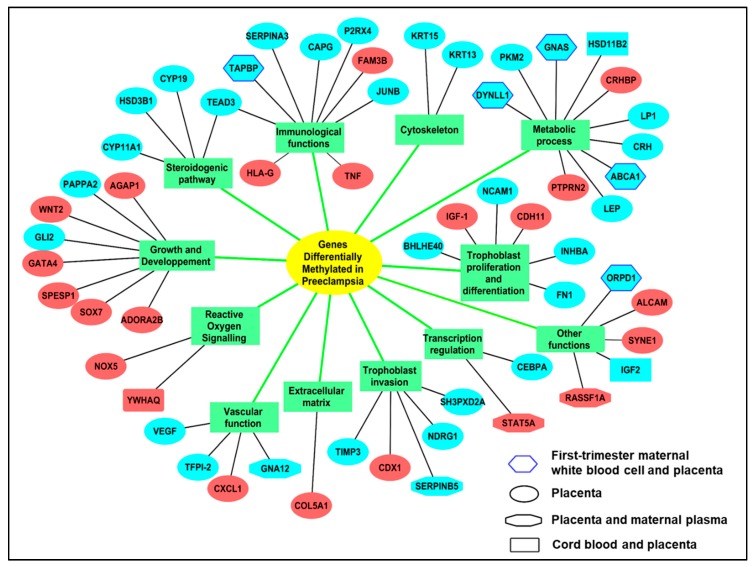
Overview of major methylation alterations in preeclampsia. The main pathways are shown in green boxes. The significant alterations in methylation may be associated either to increased or decreased gene expression (hypermethylated in red and hypomethylated in blue).

The major modifications of methylation occurring in preeclampsia are presented as Figure 3.

##### Limits of the Genome-Wide, Multicellular Approach for Preeclampsia Methylation Profiling

As mentioned earlier, a recurrent criticism of genome-wide comparisons between normal and PE placenta is linked to the fact that in general placental samples of PE patients are collected at earlier terms than controls. However, the existence of methylation profiles for control placentas throughout gestation [51] now allows to make the part between the effect of the placental ageing and the effects of the pathology per se. Other limits of these approaches are the complexity of the cell material, the variation between the degree of severity of the disease or the various statistical tests that are used in the different studies. Also, several studies have brought attention to the lack of reproducibility in high-throughput genomic, transcriptomic and epigenomic studies. This has been recently discussed by Komwar and coworkers in a recent study were they analyze the sources of variation in preeclampsia high-throughput studies an propose a methodology to ensure reproducibility and thus facilitate the integration of data across studies [198].

##### Single-Cell Analysis, the Next Frontier to Methylation Epigenomic Approaches

Gene-scale expression studies were recently carried out at the single cell scale [199] and have provided evidence of gene expression shifts during the CT, SCT and EVT differentiation steps, as well as, allowed the reconstructions of differentiation trajectories. This has also been analyzed by genome-scale DNA methylation analysis. Gamage and coworkers have analyzed by RRBS side population trophoblasts, CTs and EVTs from human first trimester placentas [56]. Forty-one genes involved in EMT and metastatic cancer pathways were found methylated between CT and EVTs, possibly contributing to the invasive phenotype of these cells. In the BeWo cell model where fusion can be induced by forskolin, RRBS analysis performed before and after fusion, showed altered methylation of genes involved in cell differentiation and commitment, together with a gain in transcriptionally active histone marks such as H3K4me3 [54]. Such approaches are for the moment difficult to transpose to placenta pathophysiology. Instead, several systems where methylation influences normal placental function have been studied. As an example, we present below the epigenetic regulation of genes involved in placental invasion and PE.

##### An Example of Specific Gene Alterations of Methylation: Regulation of Invasion

MMPs are well-characterized proteins involved in trophoblast invasion and angiogenesis during pregnancy. They constitute a family of 23 Zn^2+^ and Ca^2+^-dependent proteases that degrade the extracellular matrix. This family of proteins presents abnormal concentration and behavior in placental diseases such as PE [200], placenta accreta and placenta percreta [201,202]. This has been recently reviewed for preeclampsia [203]. A decreased level of MMP-2 and MMP-9 reduces the remodeling of spiral arteries in early gestation. Besides, other MMPs, such as MMP-1 and MMP-14, may also have a role in this disease. Epigenetic mechanisms are at work for controlling MMP gene expression.

Li and coworkers observed that TET2 is involved in the demethylation of the *MMP-9* promoter, this being associated to the downregulation of the protein and contributing to trophoblast shallow invasion [204].

TIMP3, a MMP inhibitor, shows the highest methylation reduction (over 15%) in EOPE compared to control placentas with an inverse correlation between methylation level and gene expression suggesting an increased transcription of TIMP3 in PE placentas [180,189]. Low levels of TIMP3 lead to poor invasion of the trophoblast and placenta hypoperfusion. Moreover, TIMP3 may be able to inhibit angiogenesis by blocking vascular endothelial growth factor binding to its receptor contributing to impaired placenta blood vessels development. Also, genetic variations of the gene have been associated with cardiovascular disorders and hypertension.

#### 3.1.2. Maternal Blood Epigenetic Marks in Preeclampsia

Alterations in the levels of many plasma and serum proteins have been associated with PE. In 2013, White and coworkers showed that PE was favoring hypermethylation in white blood maternal cells using the methylation-27k arrays from Illumina [205]. GRIN2b. GABRA1. PCDHB7 and BEX1 were found differentially methylated, with an enrichment of the neuropeptide signaling pathway. The re-analysis of methylation of genes known to be involved in PE revealed that in maternal circulating leukocytes, CpG sited from 4 genes associated with PE, POMC, AGT, CALCA and DDAH1, showed differential methylation in PE compared to control, with moderate methylation differences (<6%) [206]. These 4 genes are known to alter immunomodulation and inflammatory response, suggesting that at least alterations of the placental physiology in preeclampsia have epigenomic consequences on maternal circulating cells.

During pregnancy, 3 to 6% of cell-free DNA in the maternal blood plasma is derived from the placenta. Oxidative stress in PE leads to increased trophoblast apoptosis and the release of SCT microparticles and a five to ten-fold increase in circulating fetal DNA in the maternal bloodstream compared with control counterparts [207,208]. These free fetal molecules and their methylation status have been proposed as a non-invasive biomarker of fetal and placental pathologies before the onset of symptoms. This has been shown for Maspin, for which the unmethylated version have a median methylation more than 5.7 fold higher in PE than control pregnancies [209,210]. Another epigenetic marker of preeclampsia is the methylation of RASSF1A (Ras Association domain-containing protein 1) promoter [177,211,212].

#### 3.1.3. Maternal Endothelial Cells

There is limited access to maternal vessels in pregnancy, nevertheless DNA methylation was assessed from this material in 2012 using the 27K methylation array of Illumina [213]. From 14.495 genes interrogated by the array, 65 genes were identified as hypomethylated in PE. Clustering leads to identify biological processes such as smooth muscle contraction, thrombosis, inflammation, redox homeostasis, sugar metabolism and amino acid metabolism. These alterations of the maternal endothelium suggest potential effects on cardiovascular life of the mother after preeclampsia. Focusing on collagen metabolism, the authors revealed an increased expression of MMP1 and MMP8 in vascular smooth muscle cells and infiltrating neutrophils of omental arteries of preeclamptic women, which was associated with reduced methylation in the promoters of both genes in pathological patients compared to control patients [213]. In the same study, several other MMPs, showed reduced hypomethylation in PE patients albeit with lower significance [214,215]. Moreover, pregnant women under dietary supplementation may restore the reduced methylation in the promoters of these genes and be protected against the development of PE. Interestingly, all these MMPs genes are located in chromosome 11, which may be indicative of a specific sensitivity of this chromosome to epigenetic changes caused by oxidative stress during the development of the pathology. The same team reported the reduced methylation in the promoter region of TBXAS1 gene in correlation with increased gene and protein expression of thromboxane synthase in vascular smooth muscle, endothelium and infiltrating neutrophils [215]. Increased levels of thromboxane synthase induce the overproduction of thromboxane A2, a potent vasoconstrictor and platelet activator, contributing to hypertension and coagulation abnormalities classically related to PE.

#### 3.1.4. Cord Blood Cells

In 2014, Nomura and coworkers analyzed the global methylation profile of cord blood cells using the LUMA technique [161] and failed to observe an actual difference but with a limited number of controls samples (5) [216]. Genome-Wide Methylation analysis using the 450K microarray tool on neonatal cord blood DNA showed a significant genome-scale hypomethylation in neonatal cord blood DNA associated with EOPE, with 51,486 hypomethylated and 12,563 hypermethylated CpGs [187]. In this study the most differential methylated genes were associated with inflammatory pathways, cholesterol and lipid metabolism, including IL12B, FAS, PIK31 and IGF1. Deregulation of both metabolic pathways may increase the risk of cardiovascular diseases in the fetus [187]. The same microarray approach allowed to identify 5001 mostly hypermethylated regions in umbilical cord white blood cells and 869 mostly hypomethylated regions in the placenta [217]. In the cord blood cells, the gene networks enriched were involved in cardiovascular system development, cell cycle, cancer, cell morphology, infectious diseases, suggesting specific alterations that could have long-term consequences on the fetal health.

Some studies focused on mitochondrial DNA, showing hypomethylation in PE cord blood cells. The most affected loci are keys in mitochondria functionality: D-loop (control of mitochondrial DNA replication), Cytochrome C oxidase subunit 1 gene (respiratory chain) and TF/RNR1 locus (necessary for protein synthesis) [218]. Increased copy of mitochondria is observed in the placenta and maternal blood during PE suggesting an adaptive response to stress [219,220]. This is also observed in mouse models of PE [221]. Hypomethylation in the D-loop may lead to increased mitochondrial replication explaining the pathological increase of mitochondrial DNA. Methylation assay in endothelial colony-forming cells present in cord blood from PE presents differential methylation level in genes related to RNA metabolic processes, cellular protein modification processes and in positive regulation transcription, as assessed with the EPIC Illumina array, interrogating over 850,000 CpG [222]. However, at later passages, an increased number of genes are abnormally methylated. This suggests that preeclampsia may drive an altered epigenetic program in endothelial cell precursors that will be the building bricks of the newborn vascular system and program later complications.

### 3.2. Non Coding RNAs

Non-coding RNAs have been found to be differentially expressed in preeclampsia by a number of sources. Some studies have focused on investigating differential expression patterns between PE placental samples of different severities versus control groups looking for miRNAs or lncRNA [223,224,225,226], without generally identifying consensual signatures. With the aim of identifying potential biomarkers that could be used diagnostically to predict preeclampsia onset, many groups have set out instead to identify molecules differentially expressed in the plasma of patients, which could potentially be detected by mean of a simple blood test [227]. lncRNA and miRNA are the two classes of non-coding RNAs that have dominated the scene of non-coding molecules in preeclampsia. Other classes of non-coding RNAs have been identified, such as circular RNAs, that appeared recently in the context of PE development and future research will help understand the role of these molecules in the regulation of gene expression and disease [228].

#### 3.2.1. LncRNAs in Preeclampsia

Long non-coding RNAs are RNA molecules longer than 200 nucleotides which are involved in regulation of cell function through a wide range of mechanisms. lncRNAs are expressed in the nucleus as single stranded RNA molecules, which can either function in their native form or undergo maturation through the addition of a 5′cap and polyA tail; however, they are never translated into a protein product [229]. They regulate cell function by a wide range of mechanisms: alteration of the stability of target mRNAs, direct recruitment of chromatin modification enzymes, segregation of transcription factors through specific binding sites contained within the lncRNA sequence, warehousing miRNA as ‘miRNA sponges’, a function shared with circular RNAs [230]. For a complete overview please see Reference [231].

Transcriptomic analyses of placenta and decidua total RNAs allowed identifying differentially expressed lncRNAs between PE and control patients, often with a difference between EOPE and LOPE [228,232,233]. Most of these lncRNAs had been previously identified in the field of cancer research, often associated with cell proliferation, migration and invasion [234]. As mentioned above, given the parallels between the features of cancer cells and the trophoblasts during placentation such as fast proliferation of the trophoblast, migration and invasion of the maternal tissues, immunotolerance [235,236,237,238], this did not come as a surprise and has prompted extensive in vitro research to elucidate the roles of these lncRNAs in trophoblast physiology.

In the present review, we will focus on a few lncRNAs that have been well characterized: MALAT-1, MEG3, RNA-ATB. Finally, we will give a brief overview on how in PE some lncRNAs regulate gene expression by altering chromatin methylation state of their target genes, through direct recruitment of histone methyltransferases, bringing as examples PVT1, TUG1 and DIAPH2-AS1. H19 was discussed above for its important role in placental development and miRNA encoding lncRNA.

##### MALAT-1

Metastasis associated lung adenocarcinoma transcript-1 (MALAT-1) was firstly identified in lung cancer; it is a lncRNA of over 8 kb [239]. MALAT-1 normally localizes in the nucleus where it forms nuclear aggregates called speckles involved in the regulation of splicing factors availability [240]. MALAT-1 is overexpressed in placental pathologies associated with uncontrolled trophoblast invasion [241], which prompted Chen and coworkers [33] to investigate its expression in PE. Comparing RNA levels in 18 PE placentas with matched controls, MALAT-1 was found significantly downregulated in PE placentas. Overexpression and downregulation of MALAT-1 in JEG-3 regulates cell proliferation and invasion, while inhibiting apoptosis [33]. These findings suggest that MALAT-1 deregulation could lead to poor invasion of the maternal endometrium, affecting the spiral arteries’ remodeling and placenta development. Li and coworkers [242] have shown that the role of MALAT-1 is not restricted solely to the trophoblast but has a key role in regulating the angiogenesis and vascularization of the maternal decidua and fetal umbilical vasculature. MALAT-1 is expressed by mesenchymal stem cells (MSCs) in the maternal decidua and in the umbilical cord. These cells are pluripotent progenitors which are able of self-renewal and proliferation, differentiate to promote tissue regeneration, form de novo vasculature, angiogenesis and regulate immune system responses [243]. Li and coworkers (2017) observed a decreased MALAT-1 expression in MSCs from decidua and umbilical cord of preeclamptic pregnancies and set out to investigate its function in these cells. Similarly, MALAT-1 promotes proliferation and protects from apoptosis in isolated MSCs. Interestingly, coculture of MSCs with trophoblast cell line HTR-8/SVneo clearly showed how MALAT-1 overexpression could promote migration and invasion of the trophoblasts towards the MSCs layer. Coculture of the endothelial cell line HUVECs (Human Umbilical Vein Endothelial Cells) in supernatant obtained from MSCs which either expressed or had downregulated MALAT-1 showed how MALAT-1 promotes tube formation this process being dependent on Vascular Endothelial Growth Factor secretion. Finally, MALAT-1 over-expression increased the levels of the IDO protein, which activated macrophage maturation, proving its role in immune system regulation. These findings combined with the work of Chen and coworkers (2015) beautifully illustrates how MALAT-1 has a symmetric regulatory function in placentation: on the one hand, it promotes trophoblast proliferation, invasive and migratory potential and on the other hand, its expression in MSCs cells helps to attract and promote trophoblast invasion, stimulates tube formation, promotes angiogenesis and vascularization. Increase in Reactive Oxygen Species caused MALAT-1 and VEGF downregulation in MSCc exposed to oxidative stress in a dose-dependent manner [242]. MALAT-1 downregulation in preeclampsia could therefore have a huge impact on placentation and further development of the placenta over the course of gestation. It is possible that a first triggering event maybe of immunological nature, causes an increase of oxidative stress during implantation which will then alter the expression level of many targets, including MALAT-1; based on the data, this consequent deregulation would have an impact on both trophoblast and MSCs physiology, culminating in preeclampsia.

##### MEG3

Maternally Expressed 3 (MEG3) is an imprinted lncRNA which is expressed in many different cell types and tissues and acts as a tumor suppressor and is downregulated in many types of cancer. Physiologically, MEG3 acts by stabilising p53 and activating apoptotic responses [244]. Zhang and coworkers [34] analysed MEG3 RNA levels in 30 placentas from preeclamptic women, compared to 30 control samples and found a statistically significant 80% downregulation. These results were consistent with those of Yu and coworkers [245] studying a cohort of 20 preeclamptic and 20 control placentas, finding that MEG3 RNA was only 28% of the RNA levels of the control group. To elucidate in more detail the function of MEG3 in placenta, Zhang and coworkers (2015) overexpressed MEG3 in two trophoblast cell lines (JEG3 and HTR-8/SVneo), showing enhanced antiapoptotic effects, while downregulation of MEG3 increased the apoptotic cells. Analysis of protein markers showed how MEG3 downregulation increased the levels of pro-apoptotic proteins such as Caspase-3 and Bax. These results contrast with what is observed in cancer, where MEG3 expression is rather associated with the activation of proapototic pathways, possibly suggesting a different mode of action of MEG3 in these cell types. Yu and coworkers [245] focused on the link between MEG3 expression and endothelial-mesenchymal transition (EMT). During implantation and placentation, the trophoblasts undergo EMT in order to be able to migrate and invade the maternal tissues. MEG3 downregulation correlated with increased E-cadherin levels and downregulation of mesenchymal markers such as N-cadherin, vimentin, slug (encoded by the gene SNAI2), in placental RNA and protein extracts, placental sections and in vitro tests (HTR-8/SVneo trophoblast cell line). Changes in MEG3 expression did not influence proliferation but MEG3 overexpression promoted migration and trophoblasts invasion through matrigel matrixes [34,224]. Altogether, MEG3 protects from apoptosis, promotes migration and invasion by regulating endothelial-mesenchymal transition in trophoblast cells and therefore its downregulation possibly affects trophoblast invasion and placentation, playing a key role in preeclampsia. Consistently, the imprinting control region (IG-DMR) of the DLK1-MEG3 cluster was very recently found hypermethylated in human umbilical veins from preeclamptic pregnancies, with an altered expression of both imprinted genes, a lower secretion of nitrite, VEGF and a higher secretion of endothelin 1 (ET1) all factors able to mediate pathological mechanisms in the offspring from preeclampsias [246].

##### RNA-ATB

As with many other lncRNAs, lncRNA-activated by TGFβ (RNA-ATB) was first discovered in cancer, upregulated in hepatocellular carcinoma, it promotes cell proliferation, migration and invasion [247]. It has been reported that in hepatocells RNA-ATB is expressed in response to TGFβ and in fibroblasts; it can create positive feedback regulation by promoting TGFβ paracrine release. Lnc RNA-ATB was found to be significantly downregulated in placental samples from women with preeclampsia. Moreover patients with EOPE showed an even stronger deregulation [248]. Given the proliferative, invasive and migratory features of trophoblasts and in particular extravillous trophoblast, Liu and coworkers (2017) investigated lncRNA-ATB function in trophoblast cell line HTR-8/SVneo, which is a standard in vitro model of extravillous trophoblast. While overexpression of lncRNA-ATB increased the proliferative, migratory and invasive potential of HTR-8/SVneo cells, the downregulation caused a steep decrease in proliferation, migration and invasion, proving that this gene has an important role on the physiology of the extravillous trophoblast and that its deregulation could explain an aberrant implantation and endometrium invasion in preeclampsia, potentially being linked to incomplete spiral artery remodeling. Whether RNA-ATB regulates trophoblast function through the interaction with members of the miR200 family is yet to be determined. However, increased miR200 has been found to affect the development of endometrium receptivity, negatively impacting implantation [249]. In labor, miR200 is upregulated in the human uterus and has been associated with pre-term labor in murine studies [250]. It seems likely that an interaction between RNA-ATB and miR200 is required for correct placental development, gestation and delivery.

##### PVT1, TUG1 and DIAPH2-AS1: Regulating Gene Expression through Recruitment of Chromatin Remodeling Complexes

lncRNAs work through different mechanisms, depending on the specific lncRNA, the cell type, the downstream targets [231]. In the past few years a few lncRNAs have been identified in preeclampsia as potential modulators, among which PVT1, TUG1 and DIAPH2-AS1 adopt the same mechanism of action. lncRNA TUG1 is downregulated in preeclamptic placentas. Interference of TUG1 in trophoblast cell lines (JEG3 and HTR-8/SVneo) negatively affected cell proliferation and growth, migration and invasion, network formation, while it increased apoptosis [31]. Transcriptome analysis by RNA-sequencing of HTR-/SVneo cells in which TUG1 was downregulated showed a prevalence of affected genes involved in cell growth, migration and apoptosis. Xu and coworkers (2017) identified RND3 as main downstream factor involved in the phenotypic effects of TUG1 downregulation. RND3 mRNA and protein levels were strongly upregulated in response to TUG1 interference in vitro and RND3 mRNA was upregulated in preeclamptic placenta. RND3 is also known as RhoE, a GTPase that acts as a tumor suppressor, negatively regulating proliferation, migration and invasion [251]. In vitro experiments beautifully elucidated the mechanism by which TUG1 modulates RND3 expression—TUG1 directly interacts with the histone modification factor Enhancer of Zeste Homolog 2 (EZH2) and recruits it to the RND3 promoter, where EZH2 drives the silencing of RND3 by tri-methylating H3K27, resulting in strong RND3 downregulation [31]. A year later, Xu and coworkers [252] identified another lncRNA PVT1, strongly downregulated in preeclamptic placenta, whose downregulation negatively affects proliferation and increases apoptosis of trophoblast cell lines. PVT1 was found to recruit EZH2 to the promoter of the transcription factor ANGPTL4, driving its repression by increase in repressive chromatin markers: this could partially explain the phenotypic effects of PVT1 deregulation. Feng and coworkers [253] uncovered a complicated regulatory network behind PAX3 deregulation in preeclampsia which is linked with decreased proliferation, invasion and migration of trophoblast cells [254]. PAX3 is a transcription factor downregulated in preeclamptic placentas and this correlates with DNA hypermethylation of the promoter region [171,254]. In this study, Feng and coworkers (2019) found that in preeclamptic placentas lncRNA DIAPH2-AS1 is upregulated along with the transcription factor HOXD8. In vitro experiments in HTR-8/SVneo cells clarified the regulatory network: under hypoxia the transcription factor HOXD8 is upregulated and induces expression of the lncRNA DIAPH2-AS1. DIAPH2-AS1 recruits lysine-specific demethylase 1 (LSD1) to the promoter of PAX3 where it alters the chromatin modification state, decreasing methylation of Histone H3. LSD1 can also modify DNA methyl-transferase 1 (DNMT1), stabilizing it. ChIP experiments showed enrichment of LSD1 and DNMT1 at the PAX3 promoter, which correlated with increased DNA methylation and mRNA repression. Interference of DIAPH2-AS1 was enough to reverse the phenotype and increase PAX3 levels [253]. These studies underscore that different epigenetic mechanisms regulate gene expression. It is possible that certain mechanisms are favored in different cell types and future studies will help identify the conserved regulatory networks that plays a role in the etiology of preeclampsia.

#### 3.2.2. micro RNA and Preeclampsia

##### microRNAs in Preeclampsia

The first study on microRNAs (miRs) in preeclampsia was published in 2007. In this study, the expression levels of a subset of 157 miRNAs expressed in the placenta were tested by qRT-PCR in human placental samples from pregnancies without any complications, with PE, and with PE and small for gestational age (SGA) outcomes. 153 miRNA were detected in the placenta RNA samples and three of them were found to be upregulated in PE: miR-210, miR-155, miR-200b [255]. The first global transcriptomic analysis of microRNAs was performed with 20 PE placental samples and 20 controls, with microarray technology by Zhu and collaborators in 2009. Comparing gene expression profiles of the severe PE group with controls, 11 microRNAs were upregulated and 23 downregulated. Among them, many microRNAs are organized in chromosomal clusters: downregulated clusters are found in 13q31.3, 14q32.31, Xq26.2, Xq26.3, while upregulated clusters are found in 19q13.42 suggesting co-regulation profiles [256]. An integrative analysis was conducted comparing distinct datasets with the aim of identifying microRNAs–transcripts regulatory networks in preeclampsia. resulting in the construction of a map of putative microRNA-gene target interactions in developmental process, response to nutrient levels, cell differentiation, cell junction, membrane components [257].

Although many studies followed, most of them aimed at identifying differentially expressed miRs in placenta and in plasma samples from PE women. Fewer studies have focused in other cell types present in the placenta. For example, in fetal endothelial cells downregulation of miR-29a-3p and miR-29c-3p and upregulation of miR-146a is observed in PE patients [258]. Both miR-29a and miR-29c show proangiogenic functions by stimulating HUVECs proliferation and tube formation through VEGFA-induced and FGF2-induced cell migration pathways [259]. However, other studies suggest an antiangiogenic role of miR-29c through downregulation of the IGF-1 proteins at the post-transcriptional level [260,261]. On the other hand, miR-146a inhibits the de-novo formation of blood vessels in-vitro and reduces tube formation ability in HUVECs [260,261]. The study of the role that miRs may have in the different cell types present in the placenta is indispensable to understand the role of this molecules in the development of the disease. In the long term, it has also been shown that miRNA profiles in the neonate is altered following an hypertensive pregnancy; for instance the level of mir-146a at birth predict microvascular development three months later [262].

Many studies followed, aimed at identifying differentially expressed miRs in placenta and in plasma samples from PE women.

In this review, we will discuss the most well characterized microRNAs miR-210, miR-155 and give an overview of some of the research that has been carried out on circulating microRNAs, given their potential as clinically relevant biomarkers.

#### miR-210

miR-210 is a microRNA involved in the regulation of mitochondrial function and hypoxia response. It has been well characterized, in placentas as well as in different cancer and tissue types [263]. Most of the knowledge on the regulatory pathways that involve miR-210 comes from oncology research. miR-210 has been soon identified as one of the early hypoxia-response miRs, being directly regulated by the Hypoxia inducible factor 1α (HIF-1α) [264]. Under hypoxic conditions, miR-210 alters mitochondrial function promoting a metabolic switch to glycolysis. This is achieved by negative targeting of genes involved in the electron-transport chain, namely iron- sulfur cluster scaffold homolog (ISCU) and cytochrome C oxidase assembly protein (COX10). As a result, miR-210 also increases the levels of Reactive Oxygen Species (ROS) [265]. Under hypoxia, miR-210 and HIF-1α establish a positive feedback regulation that maintains expression of both factors. This is achieved by miR-210 downregulation of the mRNA of Glycerol-3-Phosphate Dehydrogenase 1-Like, which would otherwise contribute to targeting HIF1α to the proteasome for degradation. Conversely, stabilized HIF1α directly activates miR-210 expression [266].

In endothelial cells, miR-210 is involved in regulating angiogenesis and vascularization which are fundamental processes in placenta development. Hypoxia causes miR-210 activation which protects endothelial cells from apoptosis and stimulates chemotaxis driven by VEGF, migration and tube formation [267]. In preeclampsia, miR-210 was first identified as upregulated in placenta samples by Pineles and collaborators (2007) using qPCR. In the first comprehensive study carried out with microarray technologies, miR-210 was consistently found upregulated in placenta of severe preeclamptic women, however in mild preeclampsia it was found to be downregulated, which might suggest different mechanisms at play or rather different metabolic states of the placenta, with a more pronounced ischemia in severe preeclamptic placentas [256]. Moreover, subsequent analyses identified significantly upregulated miR-210 in plasma samples from patients with preeclampsia [181]. In the context of PE miR-210 is involved in the mitochondrial dysfunction observed, which causes metabolic imbalance, excessive ROS production and cell damage. Similarly to what happens in cancer, miR-210 negatively regulates ISCU which is downregulated in preeclampsia samples, directly affecting mitochondrial architecture and functionality [268,269,270]. The deregulation of miR-210 was also found in the placentas of mice from a preeclamptic model [134].

miR-210 is also an important modulator of trophoblast phisiology. In vitro studies using isolated primary trophoblasts and trophoblast cell line JAR, proved how hypoxia induces an increase in miR-210 levels. Artificial overexpression of miR-210 in JAR cells caused a significant downregulation of migration and invasion. In trophoblast cells, hypoxia and ROS can activate HIF1α but more importantly NFκ-B p50—which si found upregulated in preeclamptic placenta tissues. NFκ-B p50 binds a consensus sequence in the miR-210 promoter, activating its expression. In trophoblasts, miR-210 interacts with a perfect match with the 3′-UTR of the transcription factor homeobox-A9 (HOXA9), causing both degradation of the mRNA and downregulation of translation. Another direct target is Ephrin-A3 (EFNA3), a ligand of the Ephrin binding receptors, in this case miR-210 binds the 3′UTR of the gene with an imperfect match, causing only translational downregulation. These two transcription factors activate expression profiles involved in migration, invasion and vascularisation [181]. Therefore, in trophoblast, miR-210 expression correlates with a negative regulation of migration and invasion, mediated by downregulation of EFNA3 and HOXA9, in response to hypoxia, ROS and activated NFκ-B signaling.

Further studies have identified additional downstream targets of miR-210 in preeclampsia, which are downregulated in preeclamptic samples and whose expression is altered upon miR-210 activation in cell models. A few examples are inflammation related molecules STAT6 and IL-4 [271], potassium channel modulatory factor 1 (KCMF1) [272], thrombospondin type I domain containing 7A (THSD7A) [273].

This mounting body of evidence highlights a key role of miR-210 in the development and maintainance of a preeclamptic phenotype. However it is still not clear which is the triggering event. It is possible that complications during implantation trigger an immune response which would create a pro-inflammatory environment, activating NFκ-B signaling, causing aberrant expression of miR-210 and all consequent downstream cascades. Recently, Chen and collaborator (2019) analysed the inflammatory profile of preeclamptic women, compared to patients which experienced healthy pregnancie [274]. The concentrations of proinflammatory cytokines (IL-6, IL-17) were higher in plasma samples from peripheral blood in the preeclampsia group. Moreover, Transforming Growth Factor β1 (TGF β1) levels were higher as well. TGF β1 has the function of promoting the prevalence of a subset of regulatory T cells (Tregs) that maintain immunotolerance, allowing a successful implantation and avoiding an immune response against the foetal tissues. These Tregs are characterised by expression of the fork-head box p3 (Foxp3) transcription factor, which promotes an immunotolerant phenotype [275]. However, proinflammatory signals such as IL-6 cause the activation of T cells at the expense of Foxp3-positive Tregs, causing an activation of inflammatory responses. Zhao and coworkers showed that miR210 was upregulated in preeclamptic placentas and Foxp3 mRNA and protein levels were found downregulated, previous studies had shown evidence of direct regulation of Fox3p by miR210, suggesting a pivotal role of this microRNA in regulating the threshold of immunotolerance by altering the balance of Foxp3+ Tregs/activated Tcells [276].

#### miR-155

miR-155 is upregulated in preeclamptic placentas [255]. This upregulation correlates inversely with the level of cysteine-rich protein 61(CYR61) [277], which is a factor secreted by different cell types, including trophoblast, involved in promoting migration, invasion, angiogenesis and vascularisation [278,279]. miR-155 directly targets the 3′-UTR of CYR61 mRNA with a perfect match, causing transcriptional and translational repression. In vitro experiments (HTR-8/SVneo trophoblast cell line) showed how miR-155 inhibits CYR61-mediated expression of VEGF, inhibiting trophoblast migration [277]. Decreased trophoblast-mediated secretion of VEGF would negatively affect angiogenesis and vascularisation in the site of placenta development.

miR-155 regulates trophoblast proliferation and migration also by directly targeting the cell cycle gene Cyclin D1 [172]. Cyclin D1 is involved in cell cycle progression, migration and invasion of trophoblast lineages, downregulated in preeclamptic placentas at both mRNA and protein levels [280,281,282]. In vitro studies have shown how miR-155 through direct targeting of the 3′UTR of CyclinD mRNA downregulates mRNA and protein levels, negatively affecting migration, causing cell cycle arrest and decrease in proliferation in HTR-8/SVneo cells [42]. Exiting cell cycle is a step of terminal differentiation, which suggests how miR-155 overexpression, as found in preeclampsia, could lead to a premature differentiation of cytotrophoblasts, possibly inducing syncytialization. This phenomenon would cause depletion of the cytotrophoblast pool, accelerating placental aging.

In sum, miR-155 modulates proliferation, migration and invasion of trophoblasts and its expression can affect the phenotype of endothelial cells by negatively regulating VEGF release. miR-155 deregulation could have catastrophic consequences in placentation, deeply affecting trophoblast infiltration, vascularization and angiogenesis of the developing placenta.

#### Circulating miR-155

Maternal plasma from preeclamptic women presented significantly statistically higher levels of miR-155 [283]. In blood, microRNAs are quite stable and can travel through circulation, to be uptaken by different cell types, such as endothelial and immune cells, regulating gene expression [284]. Yang, Zhang and Ding (2017) showed how plasma levels of miR-155 positively correlate with proinflammatory cytokine interleukin-17 (IL-17) and with proteinuria and urine podocytes counts in women with preeclampsia. Similarly to miR-210, miR-155 promoter presents a binding site for NFκ-B and can be activated by this inflammation master regulator, which could suggest a similar pattern of regulation for miR-155 and pro-inflammatory factors, other than a direct interaction between these genes [285].

#### miR-155 in Endothelial Cells

Endothelial cells play a fundamental role in placentation given the copious vascularisation and angiogenesis that takes place in the maternal endometrium during placentation. In preeclampsia, pro-inflammatory factors and secreted molecules from the preeclamptic placenta produce an excessive activation of the maternal endothelium, resulting in endothelyal dysfunction, culminating in inflammation, blood pressure changes, downstream systemic effects [286]. miR-155 has been found to be downregulated in human umbelical vein endothelial cells (HUVECs) from preeclamptic women, compared to HUVECs from healthy pregnant women [287]. This downregulation correlated with an increase in Angiotensin II Receptor 1 (AT1R) and increased phosphorylation of Extracellular Signal-regulated Kinases1/2 (ERKs), identifying AT1R as direct target of miR-155 [287]. Activation of the Angiotensin II- AT1R through ERK1/2 in endothelial cells causes cell cycle arrest and initiation of senescence pathways; miR-155 depletion-dependent increase in AT1R will render endothelial cells more sensitive to blood level of Angiotensin II, promoting endothelial damage [288].

miR-155 has been implicated in regulating Nitric Oxide (NO) production in endothelial cells. NO is a potent vasodilator and reduced levels of NO have been associated with preeclampsia etiology [289,290]. In vitro studies using HUVECs proved how endothelial Nitric Oxide synthase (eNOS) mRNA is a direct target of miR-155; proinflammatory stimuli upregulate miR-155 expression in these cells in vitro, downregulating eNOS and NO production [290]. As mentioned above, microRNAs can be found in plasma and miR-155 is upregulated in plasma of women with preeclampsia [69]. microRNAs can be free in circulation or travel inside vescicles and exosomes, which can be uptaken by target cells, activating signaling pathways, affecting expression profiles [291]. Shen and collaborators (2018) elegantly showed how exosomes from plasma samples of preeclamptic patients can affect eNOS mRNA and protein levels in HUVECs [292]. In particular, treatment of HUVECs in vitro with isolated exosomes from plasma of preeclamptic patients (compared to exosomes from control group) caused a statistically significant decrease in eNOS mRNA and protein levels, which correlated with decreased NO production. When analysing the composition of the exosomes, miR-155 was found to be upregulated in the preeclamptic group. Follow up in vitro tests proved how miR-155 located in the exosomes affects eNOS regulation in endothelial cells.

#### miR-155 in Vascular Smooth Muscle Cells

In arteries and arterioles, endothelial cells are interspaced by vascular smooth muscle cells (VSMCs) which thanks to their contractile properties allow vasoconstriction and vasodilation to occur, accomodating for changes in blood pressure. VSMCs generally present a contractile phenotype characterised by elongated spindle-like morphology, high concentration of contractile filaments. In response to external stimuli, they can switch to a synthetic phenotype characterised by loss of contractility markers, rhomboid morphology, increased proliferative and migratory potential; in this state VSMCs cells lose the ability to modulate vascular resistance [293]. Phenotypic regulation of VSMCs is driven by soluble guanylate cyclase (sGC) which increases intracellular levels of guanosine monophosphate (cGMP), key messenger molecule. cGMP is the substrate of cyclic GMP-dependent protein kinase (PKG) which activates downstream signaling pathways promoting VSMCs contractile phenotype. Nitric Oxide produced by endothelial cells positively modulates sGC activity, favouring vasodilation through enhancement of the VSMCs contractile phenotype [294,295].

In the presence of proinflammatory cytokine Transforming Necrosis Factor α (TNFα), miR-155 was found to be directly activated by NFκ-B in in vitro model of VSMCs. The upregulated miR-155 directly interacts with the 3′-UTR of the mRNA of PKG1 [296] and of the β1 subunit of guanylate cyclase (sGCβ1), resulting in translational repression and mRNA degradation [297]. As a consequence of sGCβ1 downregulation, intracellular cGMP levels are strongly decreased and the downregulation of PKG1 inhibits downstream pathways [296,297]. Park and collaborators (2019) co-cultured HUVECs and VSMCs, observing higher cGMP accumulation in VSMCs, which is mediated by Ntric Oxide stimulation, produced by the endothelial cells [297]. This could be countered by ectopic miR-155 expression in VSMCs. miR-155 overexpressing in response to TNFα, mediating inhibition of the sGC/PKG pathway, causes downregulation of contractile protein markers. This results in a shift of VSMCs to a synthetic phenotype, assuming a rhomboid morphology, increasing proliferation and migration rates. Interestingly the pro-contractile effects of Nitric Oxide could be cancelled by miR-155 expression [296,297]. In placental vessels of preeclamptic placenta sGCβ1 mRNA levels are downregulated [297], given the evidence provided on miR-155 repression of the sGC/PKG pathway, we can imagine that PKG1 might be downregulated as well. In response to inflammation, both endothelial and smooth muscle cells are affected and in preeclampsia they overexpress miR-155 which alters their ability to produce and respond to vasodilation stimuli. Taken together, this evidence highlights the pivotal role of inflammation and miR-155 in the etiology of the preeclamptic phenotype.

#### Potential Biomarkers: microRNAs Circulating in Maternal Plasma

Since the identification of circulating small RNAs in plasma samples, the prospect of their potential use as diagnostic and predictive biomarkers has fueled extensive research [298]. In the context of preeclampsia, the finding that small microRNAs with placental origin can travel in the blood circulation and affect systemically different cell types opens new avenues for the understanding of the mechanisms of this complex disease [299,300].

In Table 4 are listed some of the microRNAs that have been found deregulated in plasma samples of preeclamptic patients. In several studies, groups of microRNAs differentially expressed have been analyzed for their potential as predictive biomarkers of the preeclamptic phenotype [301,302,303,304,305]. These studies show how blood levels elevation of PE-associated microRNAs can be predictive for the preeclamptic phenotype starting from the second trimester. Li and collaborators (2015) evaluated the predictive values of the upregulated micro-RNAs miR-152, miR-183 and miR-210 by plotting the corresponding receiver operating characteristic curves. In the second trimester samples, the Area Under the Curve (AUC) indicated strong predictive values and were respectively 0.93 for miR-210, 0.97 for miR-183 and 0.94 for miR-152. Interestingly, different studies investigated the predictive power of miR-210 and, even though all results highlighted its key role in preeclampsia and potential as diagnostic marker, the AUCs varied in a range between 0.7 and 0.94 [301,302,303,305]. This variation might be due to differences in patient cohorts, samples collections and handling; however, the fact that miR-210 still emerged as predictive biomarker is encouraging.

Winger and collaborators (2018) collected peripheral blood cells in preeclamptic and control patient group, analysing the expression levels of a subset of 30 microRNAs previously identified altered in preeclampsia. 48 samples were divided in a training and a validation group. Analysis of differentially expressed microRNAs in the training cohort identified a panel of 8 microRNAs with good prediction values (AUC > 0.75) and *p* value ≤ 0.05: miR-1267, miR-148a, miR-196a, miR-33a, miR-575, miR-582, miR-210, miR-16. The panel was successfully validated and the use of the 8 microRNAs combined increased the prediction power of the tests [305].

From Table 4, it is possible to appreciate the heterogeneity of findings across different studies. These discrepancies in the repertoires of circulating miRNAs complicate the identification of useful biomarkers. This heterogeneity could partly be explained by the fact that preeclampsia is a complex systemic disease that develops over months of gestation; therefore, the panel of circulating molecules in blood samples might vary considerably depending of the time point at which samples are collected. Another possible explanation might reside in the wide range of different methodologies used for the extraction of circulating RNAs which introduce technical variability [306,307]. Moreover, there is mounting evidence on how the current techniques are able to detect only a small fraction of the total bulk of circulating RNAs (WO2009093254A2). Therefore, further research is still required to improve our technical knowledge so to design better, more consistent methodologies for the identification of circulating biomarkers, that might one day allow the design of diagnostic panels for effective early detection and prevention of preeclampsia.

#### 3.2.3. Additional Considerations on the Analysis of lncRNA Functions

##### Possible Caveats of the Current Trophoblast In Vitro Models

Many of the lncRNAs found to be deregulated in preeclamptic placenta have previously been identified in cancers, where they have a role in regulating proliferation, migration, invasion and apoptosis. Most of these PE-associated lncRNAs have pro-survival and pro-migration properties, therefore downregulation is associated with activation of apoptosis, decreased migratory potential and proliferative rate.

Once they have been found to be downregulated in preeclamptic placenta, the main objective has been to investigate the molecular function of these lncRNAs in the context of placenta physiology and preeclampsia. In vitro studies have seen the use of classical cellular models of trophoblast, either choriocarcinoma cell lines (JEG3 and BeWo) or artificially immortalized cell lines (HTR-8/SVneo). Through these in vitro studies it has been established that most of these lncRNAs regulate proliferation, invasion and migration of the trophoblast.

Have we completely unfolded the role of PE-associated lncRNA in the human placenta? Since lncRNAs have been previously identified in cancers, it is possible that the functions we have attributed them in the placenta are actually a result of the fact that we are analyzing them in cell lines that are cancer-like. Therefore, there is still the possibility that these lncRNAs have additional distinct functions in placenta that could be highlighted using more physiological placenta models. The recent development of placenta organoids from stem cells rises the hope for exciting new avenues, to explore these questions [316].

##### What about the Syncytiotrophoblast?

Migration, apoptosis, invasiveness and proliferation are functions shared between cancer cells and by cytotrophoblast (CTB) especially by the extravillous trophoblast (EVT) in the placenta, the in vitro investigations into PE associated lncRNAs have so far focused on EVT cell line models (e.g., JEG3, HTR-8/SVneo). However, it is important to highlight how transcriptomic data from placenta samples are a result of overall placenta gene expression levels. The extracted placental RNA comes from all the different cell types present in the tissue and the most abundant cell populations are represented by cytotrophoblasts and syncytiotrophoblasts (SCT). Even though it is true that CTB and EVT cells are fundamental for implantation and correct placental development, the syncytiotrophoblast is the functional core of the placenta itself, constituting the barrier for nutrient exchanges between fetal and maternal vasculatures and acting as secretory organ that hormonally regulates progression of gestation. Liu and coworkers (2017), in their work on RNA-ATB, showed a strong in situ hybridization staining of lncRNA-ATB in the syncytiotrophoblast layer of the placenta, reinforcing the idea that the syncytiotrophoblast might be equally affected by deregulation in the lncRNAs species [248]. Yu and coworkers (2018) work on MEG3 showed how MEG3 downregulation observed in preeclampsia correlates with an increase in adhesion molecule E-cadherin [224]. While it is true that this molecule is important for endothelial-mesenchymal transition, and its alteration would affect trophoblast invasion and EVT migration, E-cadherin downregulation after cytotrophoblast cell-cell interaction has been implicated in CTB syncytialization [317]. Suggesting that MEG3 might affect STB physiology as well.

Therefore, there are still potentially interesting questions to be raised: what are the effects of downregulated lncRNAs on the physiology of the CTB and SCT? Do we see an alteration of the proliferative state of the CTB, does this cause premature placental aging? Does this deregulation affect the differentiation potential of the CTB, affecting the balance between CTB renewal and SCT terminal differentiation? Do these lncRNAs have other functions, exclusive to placenta, other than the ones shared with cancer?

### 3.3. Histone Modifications

Few studies addressed the question of histone code modifications in PE. Chakraborty and coll. evidenced a HIF-KDM3A-MMP12 signaling cascade that promotes trophoblast invasion and trophoblast-directed uterine spiral artery remodeling in rat placenta and human placental cells. Hypoxia drives HIF activation and KDM3A expression, which in return will alter the histone methylation status of genes promoting development of the invasive trophoblast lineage and tissue remodeling, illustrated with trophoblast-derived MMP12 activation [318]. Hypoxia was also shown to affect the histone demethylase JMJD6 (Jumonji domain containing protein 6) and JMJD6 demethylase activity was shown to be drastically reduced in PE placenta as compared to Control Placenta [319]. Very recently, the expressions of HDACs were investigated in PE placentas and only HDAC9 was found downregulated both at the mRNA and protein levels in syncytiotrophoblast cells. Knock-down of HDAC9 in HTR-8/SVneo cells inhibits trophoblast cell migration and invasion. TIMP3, an inhibitory of MMP involved in invasion and tissue remodeling, is a direct target of HDAC9, identified by ChIP and is upregulated in the absence of HDAC9 [320].

### 3.4. Imprinting

Overall, preeclampsia cannot be considered an imprinting disease, despite the fact that a recent study showed that imprinted genes are more differentially expressed in PE than other genes, with paternally expressed genes (inducing placental growth) rather down-regulated and maternally expressed genes upregulated [151]. A systematic analysis of preeclampsia placental gene expression and imprinted genes was carried out in 2017 [321], which revealed altered expression of DLX5 in human PE placentas but with a rather mild deregulation (~2 fold). To be mentioned as well, the first gene identified by positional cloning in preeclampsia, STOX1, is imprinted in specific placental cell subtypes [322,323]. The mutation originally found in STOX1 has rather a gain-of-function effect [323] and in fact, overexpression of STOX1 induces a preeclamptic expression profile and a preeclamptic phenotype in cells or in mice, respectively [7,324]. To note, however, we have no evidence that Stox1 is imprinted in mice, therefore it is suspected that the mere ectopic and untimely overexpression of this factor is the cause of the disease. The idea that an imprinted gene is implicated in preeclampsia has been cleverly substantiated by Jennifer Graves as early as 1998 [325] and she gave theoretical reasons why this should be the case. The future will tell us if more examples of imprinted preeclampsia-associated genes exist in the human genome.

## 4. Perspectives and Conclusions

The recent years have seen the emergence of an increasing number of studies focused on the role of epigenetics in the regulation of placental development and on its potential implication in placental pathologies. However, we still lack a precise picture on how these epigenetic modifications correlate with gene expression. In particular, we have a limited knowledge on how DNA-methylation or Histone modifications impact gene expression in normal and pathological placenta development. In addition, our knowledge on the mechanisms regulating the dynamics of the instauration of the different epigenetic marks across development is very scarce. Nevertheless, recent studies have started to reveal how epigenetics is involved in the regulation of important processes in placental development such as cell fate determination, *syncytialization* or EVT migration and invasion. The emergence of new technologies allowing the study of the epigenetic and transcriptomic profiles of the different cells types of the placenta will certainly greatly contribute to improve our understanding of epigenetics in placenta. Moreover, in the context of PE, to date, the studies analyzing epigenetic modifications have focused on the placenta, however the antiangiogenic and cytotoxic factors released by the PE placenta have the potential to induce epigenetics modifications in maternal target tissues (blood cells, endothelial cells). This could impact the future maternal and fetal health and deserves to be studied in detail. Overall, the comprehension of epigenetic regulation in preeclampsia both at the level of the placenta and other involved organs could provide new biomarkers and therapeutic targets to improve the management of this disease. For the moment, this has not been successfully applied as diagnostic or prognostic of preeclampsia. One explanation of this observation could be that the extraction of circulating RNAs from the plasma is still immature technologically, leading to discrepant results between various laboratories and absence of consensus in defining a panel of diagnostic miRNA. This may evolve in the future, leading to substantial exploitation of these markers in complex diseases, including preeclampsia.

## Figures and Tables

**Table 1:** Epigenetic mechanisms in placental development.

Epigenetic Mechanism	Target	Cell Type	Biological Relevance	Reference
H3K9/27me3	MMP-2, MMP-9	Human placenta	Related to trophoblasts motility and invasion	[23]
H3K4 acetylation + H3K9 methylation	Maspin	Human placenta	Negatively correlated with human trophoblasts motility and invasion	[24,25]
Acetylated H3	Pregnancy-Specific Glycoproteins	JEG-3	Inhibition of HDACs in JEG-3 cells up-regulated PSG protein and mRNA expression levels	[26]
HDAC3	GCMa	Cell Line	HDAC3 associates with the proximal GCMa-binding site (pGBS) in the syncytin promoter and inhibits its expression	[27]
Acetylation of H2A and H2B		Murine TSCs	Decreases the EMT and invasiveness of murine TSCs while maintaining their stemness phenotype	[28]
H3K4Me2; H4K20me3	Genome Wide	SCTs	H3K4Me2 co-localizes with active RNAP II in the majority of STB nuclei	[29]
H3K27me3	Genome Wide	vCT	H3K27me3 highly represented in vCT	[30]
lncRNA TUG1	RND3	HTR-8/SVneo, JEG-3	TUG1 epigenetically silences RND3 transcription by interacting with EZH2 involved in cellular proliferation, migration and invasion in trophoblasts	[31]
lncRNA RPAIN	C1q	HTR8/SVneo	Inhibition of proliferation and invasion. Inhibits C1q expression	[32]
lncRNA MALAT1		JEG-3	Regulates proliferation, migration, invasion and apoptosis	[33]
lncRNA MEG3		HTR8/SVneo and JEG-3	Regulates migration and apoptosis	[34]
lncRNA MIR503HG		JEG-3	Regulates migration and invasion	[35]
lncRNA LINC00629		JEG-3	Regulates migration and invasion	[35]
lncRNA SPRY4-IT1	HuR	HTR8/SVneo	Regulates migration and apoptosis/interferes with the β-catenin Wnt signaling	[36,37]
lncRNA H19	Binds small RNAs and proteins	vCT, JAR	Regulates proliferation and apoptosis	[38]
miR-141-3p and miR-200a-3p	Transthyretin (TTR)	syncytitialized BeWo	Inhibits TTR expression by directly binding to the 3’UTR of TTR. Regulate thyroxin uptake by the SCT	[39]
miR-34	Plasminogen activator inhibitor-1 (PAI-1), SERPINA3	JAR	Regulates invasion	[40,41]
miR-155	Cyclin D1	HTR-8/SVneo	attenuates trophoblast proliferation	[42]
miR-17_92, miR-106a_363, miR-106b_25	GCM1		attenuate differentiation of trophoblasts	[43]
miR-675	NOMO1, Igf1R	JEG3 cells	restricts trophoblast proliferation	[44]
C19MC miR cluster		HTR8/SVneo	impaired migration	[45]
methylation of gene body	DAXX	Human placenta	Loss of methylation during both vCT syncytialization to SCT and EVTs differentiation to invasive EVTs	[46]
methylation of gene promoter	APC	Human placenta and choriocarcinoma cells	trophoblast invasiveness	[47]
hypomethylated promoter	MASPIN	Human placenta	inhibits EVTs migration and invasion	[24,25,48]
Hypermethylated promoter	RASSF1A	Human placenta; JAR; JEG3	Possible role in cytotrophoblast development through its effects on ID2	[49]
Genome wide methylation	PMDs (Partially Methylated Domains)	human placenta: Chorionic Villi	genes involved in immune response, Epithelial-mesenchymal transition and inflammation	[50,51,52]
Genome wide methylation	Genome Wide	human SCTs compared to vCTs	hypomethylated SCTs compared to vCTS	[53]
Genome wide methylation	Genome Wide	BeWo and BeWo + Forskolin	DNA methylation status of numerous genes regulated at the expression level were altered by forskolin-induced fusion	[54]
Methylation	HOX genes: TLX1, HOXA10, DLX5	Human placenta	Increased methylation across gestation correlates with decreased expression. Involved in SCTs differentiation	[46]
Genome wide methylation	Genome Wide	Side-population trophoblasts, vCTs and EVTs	Each cell population has a distinctive methylome	[55,56]
Methylation	Cdx2; Eomes; Plet1; TcFap2c	Mice trophoblast stem cells (TSCs)	methylation regulates the expression of genes involved in the establishment of the TSCs	[57,58,59]
Methylation	Genome Wide	Blastocyst	hypomethylation of the trophectoderm compared to the inner cell mass	[60]

**Table 2:** Summary of DNA methylation studies in developing placenta using genome-wide approaches.

Sample	Method	GEO ID	Findings	Reference
First-trimester and term placenta and maternal blood	Illumina HM450		2944 hypermethylated CpG sites in the first and 5218 in third trimester placenta.	[62]
First-trimester placenta and maternal blood	MeDIP-Seq and Illumina HM450		3759 CpG sites in 2188 regions were differentially methylated	[63]
Placenta (first, second and third trimester)	Illumina HM450 and MethylC-Seq & RNA-Seq	GSE39777	Identification of partially methylated domains (PMDs) and differences between placenta and other tissues	[51]
Placenta (first, second and third trimester)	Illumina HM27		Increase in overall genome methylation observed from first to third trimester.	[64]
Term placenta	MeDIP + custom microarray		Tissue-specific differentially methylated regions in the placenta	[65]
Various human trophoblast populations	Illumina HiSeq 2000	GSE109682	Human trophoblasts are different from somatic cells in terms of global CpG methylation	[56]
Methylation profiles of E18.5 term placenta of WT and Hltf−/− mouse	Illumina HiSeq 2000 (Mus musculus)	GSE114145	Hltf-gene deletion alters the epigenetic landscape of the placenta.	[66]
Fetal placental tissue of both sexes in GR+/+ vs. GR+/− mice	Illumina HiSeq 2000	GSE123188	GR mutation in mice changes the epigenome of placental tissue in a sex-specific manner	[67]
Human placentas	Illumina HM450	GSE108567	Adjusting for batch effects in DNA methylation	[68]
Epigenetic mechanism of mouse embryo development	Illumina HiSeq 2500 (Mus musculus)	GSE104243	H3K27me3 and DNA methylation in extraembryonic and embryonic lineages	[69]
Samples from different normal human tissues	Illumina HM450	GSE103413	Identifying candidate imprinted genes	Database, unpublished
Bisulphite and oxidative bisulphite converted placental DNA	Illumina HM450	GSE93429	Hydroxymethylcytosine and methylcytosine profiles in the human placenta	[70]
Methylation in first and third trimester placental samples	Illumina Genome Analyzer Iix	GSE98752	Complex Association between DNA Methylation and Gene Expression	[71]
DNA Methylation in Human Fetal Tissues and Human IPSC	Illumina HM450	GSE76641	DNA methylation and transcriptional trajectories in human development.	[72]
DNA methylation of fetal membranes, trophoblasts and villi 2nd trimester	Illumina HM450	GSE98938	Genome-scale fluctuations in the cytotrophoblast epigenome	Database, unpublished
Developing mouse placenta	Illumina HiSeq 2000	GSE84350	DNA Methylation Divergence and Tissue Specialization in the Developing Mouse Placenta	[73]
Villous cytotrophoblasts samples	Illumina HM450	GSE93208	DNA methylation profiling of first trimester villous cytotrophoblasts	[52]
Placental tissue collected at term.	Illumina HM450	GSE71719	DNA methylation and hydroxymethylation assessment.	[74]
DNA from chorionic villus from the 1st trimester and maternal blood cell samples	Illumina HiSeq 2000 (Homo sapiens)	GSE58826	DNA Methylation Predictors of Gene Expression in the 1st Trimester Chorionic Villus	Database, unpublished
Methylation patterns of human placenta, blood neutrophils and somatic tissue	Illumina HiSeq 2000 (Homo sapiens)	GSE59988	The human placenta exhibits a dichotomized DNA methylation pattern compared to somatic tissues	[75]
mRNA and DNA methylation profiling of Dnmt3a/3b-null trophoblasts	Illumina HiSeq 2000 (Mus musculus)	GSE66049	Maternal DNA methylation in early trophoblast development	[76]
Imprinted differentially methylated regions in hu-man villous trophoblast and blood samples	Illumina MiSeq (Homo sapiens)	GSE76273	Polymorphic imprinted methylation in the human placenta	[77]
Placental villous explant culture in different growth conditions	Illumina HM450	GSE60885	Genome-wide DNA methylation identifies trophoblast invasion-related genes.	[78]
Trophoblast methylation in NLRP7 knockdown	Illumina HM450	GSE45727	NLRP7 alters CpG methylation	[79]
Bisulphite converted DNA	Illumina HumanMethylation27 BeadChip	GSE36829	Epigenome analysis of placenta samples from newborns	Database, unpublished
First trimester, second trimester and full-term placentas	Illumina HumanMethylation27 BeadChip	GSE31781	Widespread changes in promoter methylation profile in human placentas.	[80]
Chorionic villus and maternal blood cell samples	Illumina HumanMethylation27 BeadChip	GSE23311	DNA Methylation Analysis in Human Chorionic Villus and Maternal Blood Cells	[81]

**Table 3:** Differentially methylated genes in preeclampsia.

Cell Type	Gene	Methylation State in PE	Possible Target	Reference
Placenta and maternal plasma	*SERPINB5*	Hypomethylated	Trophoblast Invasion	[177]
First-trimester maternal white blood cell and placenta samples	*ABCA1*	Hypomethylated	Cholesterol transporter in macrophages	[178,179]
First-trimester maternal white blood cell, placenta samples, umbilical cord blood	*GNAS*	Hypomethylated	Diabetes, hypertension and metabolic diseases	[178,179]
First-trimester maternal white blood cell and placenta samples	*TAPBP*	Hypomethylated	Peptide loading in the Histocompatibility complex	[178]
First-trimester maternal white blood cell and placenta samples	*DYNLL1*	Hypomethylated	Phosphate metabolic processing	[178]
First-trimester maternal white blood cell and placenta samples	*ORPD1*	Hypomethylated	Opioid Receptor	[178]
Placenta	*TIMP3*	Hypomethylated	Metalloprotease Inhibitor	[180]
Placenta	*P2RX4*	Hypomethylated	Apoptosis and Inflammation	[170]
Placenta	*PAPPA2*	Hypomethylated	Insuline-like growth factor regulator	[170]
Placenta	*DLX5*	Hypomethylated	Trophoblast proliferation and differentiation	[181]
Placenta	*KRT15*	Hypomethylated	Cytoskeleton	[182]
Placenta	*SERPINA3*	Hypomethylated	Inhibition of inflammation, pathogen degradation and tissue remodeling	[183]
Placenta	*FN1*	Hypomethylated	Cell adhesion, trophoblast proliferation, differentiation and apoptosis	[182]
Placenta	*TEAD3*	Hypomethylated	Cell homeostasis, Inflammation, Coagulation, complement activation	[184]
Placenta	*JUNB*	Hypomethylated	TNF signaling pathway	[182]
Placenta	*PKM2*	Hypomethylated	Cellular metabolism	[182]
Placenta	*NDRG1*	Hypomethylated	Trophoblast invasion	[182]
Placenta	*BHLHE40*	Hypomethylated	Inhibition of trophoblast differentiation	[171]
Placenta	*INHBA*	Hypomethylated	Inhibition of trophoblast differentiation	[171]
Placenta	*CYP11A1*	Hypomethylated	Trophoblast autophagy and steroidogenic pathway	[184]
Placenta	*HSD3B1*	Hypomethylated	Steroidogenic pathway	[184]
Placenta	*TEAD3*	Hypomethylated	Steroidogenic pathway	[184]
Placenta	*CYP19*	Hypomethylated	Steroidogenic pathway	[184]
Placenta	*CRH*	Hypomethylated	Cortisol bioavailability in the placenta	[184]
Placenta	*TFPI-2*	Hypomethylated	Block in endothelial dysfunction	[185]
Placenta	*VEGF*	Hypomethylated	Angiogenesis	[186]
Umbilical cord blood, placenta samples	*IGF2*	Hypomethylated	Embryonic development and fetal growth	[179,187]
Placenta and Peripheral Blood	*GNA12*	Hypomethylated	Blood pressure	[188]
Placenta	*CAPG*	Hypomethylated	Macrophage function	[189]
Placenta	*GLI2*	Hypomethylated	Embryo development	[189]
Placenta	*KRT13*	Hypomethylated	Cytoskeleton	[189]
Placenta	*LEP*	Hypomethylated	Cell homeostasis and metabolism	[190]
Placenta	*LP1*	Hypomethylated	Lipid metabolism	[191]
Placenta	*CEBPα*	Hypomethylated	Transcription stimulation of LEP promoter	[191]
Placenta	*SH3PXD2A*	Hypomethylated	Trophoblast invasion and podosome formation	[191]
Placenta	*NCAM1*	Hypomethylated	Trophoblast-trophoblast interactions and adhesion	[174]
Cord blood samples	*HSD11B2*	Hypomethylated	Cortisol transmission from the mother to the fetus	[192]
Placenta	*WNT2*	Hypermethylated	Placentation and cell signaling	[158,193]
Placenta	*SPESP1*	Hypermethylated	Fertilization	[158]
Placenta	*NOX5*	Hypermethylated	Reactive Oxygen Species signaling	[158]
Placenta	*ALCAM*	Hypermethylated	Cell Adhesion	[158]
Placenta	*IGF-1*	Hypermethylated	Placentation, trophoblast function, fetal growth.	[194]
Placenta	*SOX7*	Hypermethylated	Embryonic development and cell fate	[155]
Placenta	*CDX1*	Hypermethylated	Trophoblast invasion restriction	[155]
Placenta	*CXCL1*	Hypermethylated	Chemokine inducer of angiogenesis	[155]
Placenta	*ADORA2B*	Hypermethylated	Placenta impairment and fetal growth restriction	[155]
Placenta	*FAM3B*	Hypermethylated	Cytokine activity	[182]
Placenta	*SYNE1*	Hypermethylated	Nuclear organization and structural integrity	[182]
Placenta	*AGAP1*	Hypermethylated	Cellular development, assembly and function	[182]
Placenta	*CRHBP*	Hypermethylated	Cortisol bioavailability in the placenta	[190]
Placenta and maternal blood	*STAT5A*	Hypermethylated	Transcription activation	[195]
Placenta and maternal plasma	*RASSF1A*	Hypermethylated	Tumor suppressor gene	[177]
Placenta	*PTPRN2*	Hypermethylated	Phosphate metabolic processing	[173]
Placenta	*GATA4*	Hypermethylated	Placenta Growth	[173]
	*YWHAQ*	Hypermethylated	Cellular response to reduce oxygen levels	[196]
Placenta	*TNF*	Hypermethylated	MMP-9 stimulation, Immune system activation, cell survival, migration and differentiation	[174]
Placenta	*COL5A1*	Hypermethylated	Extracellular matrix	[174]
Placenta	*CDH11*	Hypermethylated	Trophoblast anchoring to the decidua, syncytiotrophoblast differentiation	[174]
Placenta	*HLA-G*	Hypermethylated	Maternal Immune tolerance and immune rejection	[197]

**Table 4:** Deregulated miRNA in preeclampsia.

microRNA	PE Placenta	PE Plasma	Function	Gene targets	AUC	References
miR-214	DOWN					[308]
miR-152		DOWN				[300]
miR-218	DOWN					[308]
miR-590	DOWN					[308]
miR-18a	DOWN	DOWN	Promoting trophoblast migration	SMAD2		[225,308]
miR-19a	DOWN					[308]
miR-19b1		DOWN	TGFβ-signaling	SMAD factors		[225]
miR-379	DOWN					[308]
miR-411	DOWN					[308]
miR-195	DOWN					[308]
miR-223	DOWN					[308]
miR-363	DOWN					[308]
miR-542-3p	DOWN					[308]
miR-144		DOWN	Ischemia, hypoxia			[225]
miR-15b		DOWN	Angiotensin-renin system			[225]
miR-181a	UP	UP				[225,308]
miR-584	UP					[308]
miR-30a-3p	UP					[308]
miR-151	UP					[308]
miR-31	UP					[308]
miR-210	UP	UP		PTPN2	0.7 < AUC < 0.9	[225,255,300,302,303,305,308,309]
miR-17-3p	UP					[308]
miR-193b	UP					[308]
miR-638	UP					[308]
miR-525	UP					[308]
miR-515-3p	UP					[308]
miR-519e	UP					[308]
miR-517-5p	UP	UP			AUC = 0.7	[304]
miR-518b	UP	UP				[225,304,308]
miR-524	UP					[308]
						
miR-296	UP					[308]
miR-362	UP					[308]
miR-574-5p		UP			AUC > 0.7	[302]
miR-1233-3p		UP			AUC > 0.6	[302]
miR-155		UP			AUC > 0.7	[225,303]
miR-1267		UP			AUC > 0.8	[305]
miR-148a		UP	Immune response	HLA-G	AUC > 0.9	[305,310]
miR-196a		UP			AUC = 1	[305]
miR-33a		UP			AUC = 1	[305]
miR-575		UP			AUC > 0.9	[305]
miR-582		UP	Trophoblast invasion, migration	VEGF	1	[305,311]
miR-152	UP	UP	Immune response	HLA-G	AUC > 0.9	[256,301,312]
miR-183	UP	UP	Cell differentiation, apoptosis, invasion		AUC > 0.9	[255,301,313]
miR-215		UP				[225]
miR-650		UP				[225]
miR-21	UP	UP	Apoptosis			[225,314]
miR-29a		UP				[225]
miR-300		UP	Trophoblast differentiation	ETS-1		[315]

Annotations: AUC = Area Under the Curve; SMAD2 = Mothers Against Decapentaplegic Homolog 2; PTPN2 = Tyrosine-protein phosphatase non-receptor type 2; HLA-G = Histocompatibility antigen, alpha chain G; VEGF = Vascular endothelial growth factor; ETS-1 = E26 oncogene homolog 1; TGFβ = Tumor growth factor β.

## Supplementary Materials

Supplementary materials can be found at https://www.mdpi.com/1422-0067/20/11/2837/s1. Table S1. High-throughput studies analyzing methylation profiles of different relevant tissues in the context of preeclampsia [42,43,47,54,55,56,57,58,59,60,61,62,63,64,65,66,67,68,69,70,71,72,73].
