# Supplementary material for: The Role of Epigenetics in Placental Development and the Etiology of Preeclampsia
Source: Int J Mol Sci. 2019 Jun 11;20(11):2837. doi: 10.3390/ijms20112837 (PMC6600551; doi:10.3390/ijms20112837)
Supplement: Supplementary file 1 [file ijms-20-02837-s001.pdf]

Table 1. High-throughput studies analyzing methylation profiles of different relevant tissues in the context of preeclampsia [42,43,47,54–73].

Sample	Method	GEO ID	Findings	Reference
First-trimester placenta and maternal blood (three of each) Term placenta and maternal blood (two of each)	Illumina HM450		Identified 2944 and 5218 hypermethylated CpG sites in the first and third trimester placenta, respectively that were fetal-specific and found an overlap of 2613 differentially methylated sites between maternal blood and placenta tissue	[54]
First-trimester placenta and maternal blood (14 of each)	MeDIP-Seq and Illumina HM450		Using both assays, 3759 CpG sites in 2188 regions were differentially methylated between maternal blood and placenta	[55]
Placenta (five first, ten second, 21 third trimester) for 450K Three term placentas for MethylC-Seq	Illumina HM450 and MethylC-Seq & RNA-Seq	GSE39777	Identified partially methylated domains (PMDs) cover 37% of the placental genome. RNA-seq revealed that genes with PMDs are repressed. 450K data showed that PMDs are conserved throughout gestation	[42]
Placenta (18 first, ten second, 14 third trimester)	Illumina HM27		An increase in overall genome methylation observed from first to third trimester. First-, second- and third-trimester cluster separately on a dendrogram	[56]
Term placenta (3)	MeDIP + custom microarray		Identified tissue-specific differentially methylated regions in the placenta	[57]
side-population trophoblasts, cytotrophoblasts, extravillous trophoblasts 12	Illumina HiSeq 2000	GSE109682	Human trophoblasts are primarily distinguished from somatic cells by differences in the pattern rather than the degree of global CpG methylation	[47]
Placental DNA methylation profiles of E18.5 term placenta from three wild type control and three Hltf null mouse samples	Illumina HiSeq 2000 (Mus musculus)	GSE114145	Hltf-gene deletion alters the epigenetic landscape of the placenta.	[58]
Fetal placental tissue of both sexes in GR+/+ vs. GR+/- c57/bl6 mice	Illumina HiSeq 2000	GSE123188	GR mutation in mice changes the genome-wide epigenome of placental tissue in a highly sex-specific manner	[59]

30 human placentas	Illumina HumanMethylation450 BeadChip	GSE108567	Adjusting for batch effects in DNA methylation microarray data, a lesson learned	[60]
comprehensive epigenetic mechanism of mouse embryo development and the corresponding lineage development process,	Illumina HiSeq 2500 (Mus musculus)	GSE104243	Distinct distribution of H3K27me3 and DNA methylation stabilizes the segregation of extraembryonic and embryonic lineages	[61]
67 samples from different normal human tissues	Illumina HumanMethylation450 BeadChip	GSE103413	Mining novel candidate imprinted genes using genome-wide methylation screening and literature review	Database, not published
Bisulphite and oxidative bisulphite converted DNA from 4 human placenta	Illumina HumanMethylation450 BeadChip	GSE93429	Comparison of ox-BS-450k 5-hydroxymethylcytosine and BS-450k 5-methylcytosine profiles in the human placenta	[62]
DNA methylation comparison in human placenta between 6 first trimester and 5 third trimester samples	Illumina Genome Analyzer Iix	GSE98752	A Complex Association between DNA Methylation and Gene Expression in Human Placenta at First and Third Trimesters	[63]
DNA Methylation Barcodes in Human Fetal Tissues and Human Induced Pluripotent Stem Cells	Illumina HumanMethylation450 BeadChip	GSE76641	DNA methylation and transcriptional trajectories during human development and reprogramming of isogenic pluripotent stem cells	[64]
DNA methylation profiling of amnion, basal plate, chorion, trophoblast, and villi in 2nd trimester and at term	Illumina HumanMethylation450 BeadChip	GSE98938	Genome-scale fluctuations in the cytotrophoblast epigenome over gestation and in placental pathologies	Database, not published
	Illumina HiSeq 2000	GSE84350	DNA Methylation Divergence and Tissue Specialization in the Developing Mouse Placenta	[65]
villous cytotrophoblasts samples isolated ex vivo from placental chorionic villi before they first come into contact with maternal blood (8-10 weeks of gestation, n = 9) and after (12-14 weeks of gestation, n = 10)	Illumina HumanMethylation450 BeadChip	GSE93208	DNA methylation profiling of first trimester villous cytotrophoblasts	[43]
23 placental tissue collected at term.	Illumina HumanMet	GSE71719	DNA methylation and hydroxymethylation assessment	[66]

	hylation450 BeadChip		through 450K analysis of paired bisulfite and oxidative-bisulfite conversion.	
DNA from 6 chorionic villus samples from the 1st trimester (from 3 male and 3 female fetuses) as well as 3 maternal blood cell samples	Illumina HiSeq 2000 (Homo sapiens)	GSE58826	DNA Methylation Predictors of Gene Expression in the 1st Trimester Chorionic Villus	Database, not published
We sought to compare genome-wide methylation patterns of human placenta with blood neutrophils, a homogenous somatic tissue (11)	Illumina HiSeq 2000 (Homo sapiens)	GSE59988	The human placenta exhibits dichotomised DNA methylation pattern compared to homogenous somatic tissue	[67]
mRNA and DNA methylation profiling of Dnmt3a/3b-null trophoblast	Illumina HiSeq 2000 (Mus musculus)	GSE66049	Maternal DNA methylation regulates early trophoblast development	[68]
151 assays for imprinted differentially methylated regions in human placental villous, trophoblast and whole blood samples	Illumina MiSeq (Homo sapiens)	GSE76273	Pervasive polymorphic imprinted methylation in the human placenta	[69]
17 samples of placental villous explant cultre exposed to different growth conditions	Illumina HumanMet hylation450 BeadChip	GSE60885	Genome-wide DNA methylation identifies trophoblast invasion-related genes: Claudin-4 and Fucosyltransferase IV control mobility via altering matrix metalloproteinase activity	[70]
Bisulphite converted DNA from 3 replicates of untransduced, scrambled control and NLRP7 knockdown in the undifferentiated and BMP differentiated groups	Illumina HumanMet hylation450 BeadChip	GSE45727	NLRP7 promotes trophoblast lineage differentiation, YY1 binding and alters CpG methylation	[71]
Bisulphite converted DNA from the 48 samples	Illumina HumanMet hylation27 BeadChip	GSE36829	Epigenome analysis of placenta samples from newborns	Database, not published
42 samples, with 18 first trimester, 10	Illumina HumanMet	GSE31781	Evidence for widespread changes in promoter	[72]

second trimester, 14 full term placenta	hylation27 BeadChip		methylation profile in human placenta in response to increasing gestational age and environmental/stochastic factors.	
12 human chorionic villus samples and 12 human maternal blood cell samples	Illumina HumanMet hylation27 BeadChip	GSE23311	DNA Methylation Analysis in Human Chorionic Villus and Maternal Blood Cells	[73]
